# *Zac1 *functions through *TGFβII *to negatively regulate cell number in the developing retina

**DOI:** 10.1186/1749-8104-2-11

**Published:** 2007-06-08

**Authors:** Lin Ma, Robert Cantrup, Annie Varrault, Dilek Colak, Natalia Klenin, Magdalena Götz, Sarah McFarlane, Laurent Journot, Carol Schuurmans

**Affiliations:** 1 IMCH, HBI, University of Calgary, T2N 4N1, Canada; 2 Institut de Génomique Fonctionnelle, Montpellier, France; 3 Institute of Stem Cell Research, GSF, München, Germany

## Abstract

**Background:**

Organs are programmed to acquire a particular size during development, but the regulatory mechanisms that dictate when dividing progenitor cells should permanently exit the cell cycle and stop producing additional daughter cells are poorly understood. In differentiated tissues, tumor suppressor genes maintain a constant cell number and intact tissue architecture by controlling proliferation, apoptosis and cell dispersal. Here we report a similar role for two tumor suppressor genes, the *Zac1 *zinc finger transcription factor and that encoding the cytokine TGFβII, in the developing retina.

**Results:**

Using loss and gain-of-function approaches, we show that *Zac1 *is an essential negative regulator of retinal size. *Zac1 *mutants develop hypercellular retinae due to increased progenitor cell proliferation and reduced apoptosis at late developmental stages. Consequently, supernumerary rod photoreceptors and amacrine cells are generated, the latter of which form an ectopic cellular layer, while other retinal cells are present in their normal number and location. Strikingly, *Zac1 *functions as a direct negative regulator of a rod fate, while acting cell non-autonomously to modulate amacrine cell number. We implicate TGFβII, another tumor suppressor and cytokine, as a *Zac1*-dependent amacrine cell negative feedback signal. TGFβII and phospho-Smad2/3, its downstream effector, are expressed at reduced levels in *Zac1 *mutant retinae, and exogenous TGFβII relieves the mutant amacrine cell phenotype. Moreover, treatment of wild-type retinae with a soluble TGFβ inhibitor and TGFβ receptor II (TGFβRII) conditional mutants generate excess amacrine cells, phenocopying the *Zac1 *mutant phenotype.

**Conclusion:**

We show here that *Zac1 *has an essential role in cell number control during retinal development, akin to its role in tumor surveillance in mature tissues. Furthermore, we demonstrate that *Zac1 *employs a novel cell non-autonomous strategy to regulate amacrine cell number, acting in cooperation with a second tumor suppressor gene, *TGFβII*, through a negative feedback pathway. This raises the intriguing possibility that tumorigenicity may also be associated with the loss of feedback inhibition in mature tissues.

## Background

Tissues and organs are genetically programmed to achieve their optimal, mature size, defined by total cell number and individual cellular dimensions. Several regulatory strategies are employed to control cell number, including: direct negative regulators, which inhibit alternative cell fates but permit (or instruct) a primary fate; negative feedback pathways, acting as cell sensors that halt the continued genesis of specific cell types once a feedback signal reaches threshold levels; and cell counting mechanisms, whereby the number of times a progenitor divides before differentiating is genetically determined [1,2]. In the vertebrate retina, negative feedback pathways are used recurrently for cell number control. The retina is composed of one glial and six neuronal cell types that are present in stereotyped proportions in each vertebrate species [3-5]. Based on lineage tracing, all retinal cell types are derived from multipotent progenitor cells [6-11], although distinct cell lineages likely also exist [1,12]. In mouse, retinal ganglion cells (RGCs), horizontal cells, cone photoreceptors and amacrine cells are primarily generated during the second half of the embryonic period, while rod photoreceptor, bipolar and Müller glial cell production ends on postnatal days (P) 5–6 in the central retina [3]. Differentiated RGCs, amacrine cells and cones secrete signals negatively regulating production of additional cells of that type [13-16]. However, only signals limiting production of RGCs have been identified, including Sonic hedgehog (Shh) and growth and differentiation factor-11 (GDF11) [17]. GDF11, a transforming growth factor (TGF)β family member, has similar autoregulatory functions in other tissues, including the olfactory epithelium [18] and pancreas [19], while a related molecule, GDF8 (myostatin), negatively regulates skeletal muscle mass [20], suggesting a common role for these cytokines in cell number control.

We identified *Zac1 *(*zinc finger protein that regulates apoptosis and cell cycle arrest *or *pleiomorphic adenoma gene-like 1 *(*Plag-l1*)) [21] in a screen designed to isolate genes involved in neural fate specification [22]. *Zac1 *encodes a seven-C_2_H_2 _zinc finger protein that acts as a transcriptional activator or repressor [21]. *Zac1 *is a known tumor suppressor gene and is frequently lost in multiple carcinomas [21]. *Zac1 *is also maternally repressed through genomic imprinting, a mode of epigenetic control common to many genes regulating embryonic growth. Recently, a *Zac1 *null mutation was shown to lead to intrauterine growth restriction, consistent with the kinship theory that paternally expressed genes are growth promoting [23]. However, growth retardation was not expected if *Zac1 *has tumor suppressor properties, promoting cell cycle exit and apoptosis [21,24]. We therefore examined *Zac1 *function at the cellular level, focusing on the developing retina, where it is robustly expressed [25]. Notably, in our initial cross-species studies in *Xenopus*, murine *Zac1 *unexpectedly promoted proliferation [26]. Herein we describe intra-species loss- and gain-of-function assays in mouse that in contrast reveal tumor suppressor-like properties for *Zac1 *in the retina. *Zac1 *is required to induce cell cycle exit and apoptosis at late developmental stages, with *Zac1 *mutant retinae becoming hypercellular, containing supernumary rod photoreceptors and amacrine cells. Strikingly, *Zac1 *negatively regulates rod and amacrine cell numbers through distinct autonomous and cell non-autonomous (TGFβII-mediated) inhibitory mechanisms, respectively.

## Results

### Biphasic expression of *Zac1 *in retinal progenitors and postmitotic cells

**Figure 1:**
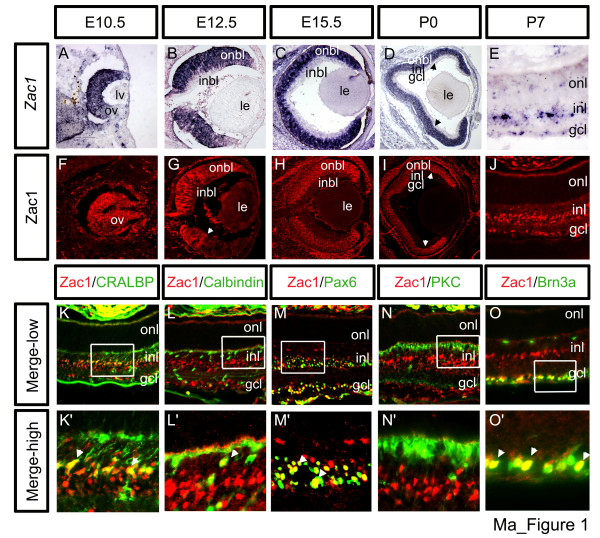
Biphasic Zac1 expression in the retina. *Zac1 ***(a-e) **transcript and protein **(f-j) **distribution from E10.5 to P7. Arrowheads in (d,g,i) mark limits of higher expression domains. **(k-o) **Identification of Zac1+ P7 retinal cells. Co-labeling with Zac1 (red) and CRALBP (green **(k,k')**), calbindin (green **(l,l')**), Pax6 (green **(m,m')**), PKC (green **(n,n')**) and Brn3a (green **(o,o')**). High magnification images of boxed areas are shown in (k'-o'). Arrowheads mark double^+ ^cells. Of 2,154 Zac1^+ ^cells analyzed, 1,238 CRALBP/Zac1 double^+ ^Müller glia; 29 calbindin/Zac1 double^+ ^horizontal cells (based also on morphology), 480 Pax6/Zac1 double^+ ^amacrine cells (in the INL) and 407 Brn3a/Zac1 double^+ ^RGCs were identified. GCL, ganglion cell layer; inbl, inner neuroblast layer; INL, inner nuclear layer; le, lens; lv, lens vesicle; onbl, outer neuroblast layer; ONL, outer nuclear layer; ov, optic vesicle.

We identified *Zac1 *in a subtractive screen designed to identify regulators of neuronal fate specification [22]. In an initial expression survey, we noted high *Zac1 *expression in the developing retina [25]. A detailed spatiotemporal characterization from embryonic day (E) 10.5 through P0 revealed high levels of *Zac1 *transcripts (Figure 1a–d) and protein (Figure 1f–i) in the outer neuroblast layer (onbl), where proliferating progenitors reside, and not in the inner neuroblast layer (inbl) of postmitotic cells that, prior to P0, primarily includes RGCs and amacrine cells (Additional data file 1 (a–c)). Confirming Zac1 expression in dividing cells, a large number of Zac1^+ ^cells incorporated the S-phase label bromodeoxyuridine (BrdU) after a 30 minute pulse at E15.5 (Additional data file 1 (d–f)). Notably, *Zac1 *expression declined in central, more mature retinal progenitors by P0 (Figure 1d, i).

At P2 (not shown), P7 (Figure 1e, j) and P21 (Additional data file 1 (g,h)), *Zac1 *transcripts and protein were detected in scattered postmitotic cells in the inner nuclear layer (INL) and RGC layer (GCL; Figure 1k–o). Double immunolabeling with cell type-specific markers at P7 revealed Zac1 expression in CRALBP^+ ^Müller glia (64.1% ± 6.26% Zac1^+^cells; n = 3 retinae; Figure 1k,k'), syntaxin^+ ^(not shown) and Pax6^+ ^amacrine cells (17.5% ± 3.6%; Figure 1m,m'), Brn3a^+ ^RGCs (17.2% ± 5.0%; Figure 1o,o') and calbindin^+ ^horizontal cells (1.2% ± 0.7%; Figure 1l,l'). Zac1 was not detected in protein kinase C (PKC)-expressing bipolar cells (Figure 1n,n') or in rod and cone photoreceptors in the outer nuclear layer (ONL).

Zac1 is thus expressed biphasically in the retina, initially in dividing retinal progenitors and later in Müller glia, RGCs, amacrine and horizontal cells.

### *Zac1 *mutants develop hypercellular retinae containing an ectopic cellular layer

To investigate the in vivo requirement for Zac1, we analyzed embryos with a Zac1 null allele [23]. Because Zac1 is maternally imprinted, Zac1+m/- heterozygotes inheriting a wild-type allele from their mother are effectively mutant for Zac1. Indeed, imprinting occurs in the gametes, and complete methylation of Zac1 is achieved in 96.8% of mature oocytes [27]. Accordingly, Zac1+m/- retinae were devoid of Zac1 immunolabeling (Additional data file 2) and were thus considered equivalent to null mutants throughout this study.

**Figure 2:**
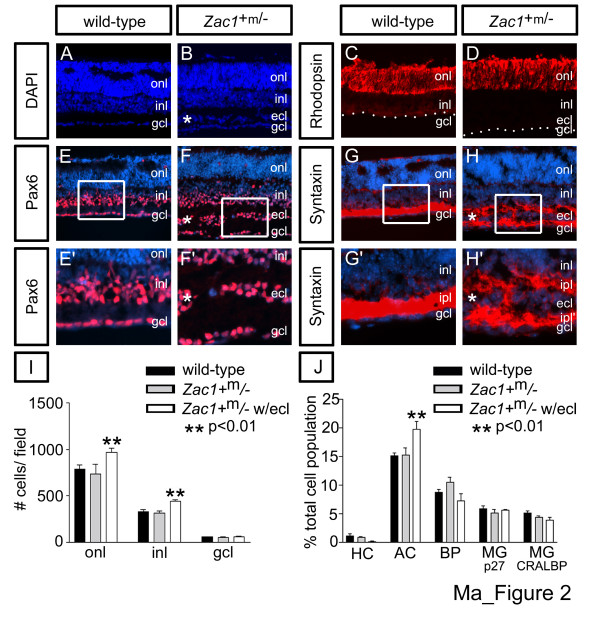
*Zac1*^+m/- ^retinae develop an ectopic amacrine cell layer and supernumerary rod photoreceptors. **(a-h) **E18.5→8DIV retinal explants. DAPI-stained **(a) **wild-type and **(b) ***Zac1*^+m/- ^explants. Rhodopsin expression in **(c) **wild-type and **(d) ***Zac1*^+m/-^+ ECL retinae. **(e,e',f,f') **Pax6 and **(g,g',h,h') **syntaxin expression in amacrine cells in wild-type (e,e',g,g') and *Zac1*^+m/-^+ECL (f,f',h,h') retinae. Asterisks mark the ECL. The duplicated IPL is labeled by ipl' in (h'). Blue is DAPI counterstain. **(i) **Average of the absolute number of DAPI^+ ^nuclei/layer in a standard counting field in wild-type (black bar; total DAPI^+ ^nuclei counted in 30 fields; ONL: 23,700; INL: 9,870; GCL: 1,776), *Zac1*^+m/- ^without an ECL (grey bar; total DAPI^+ ^nuclei counted in 9 fields; ONL: 6,615; INL: 2,826; GCL: 498 nuclei) and *Zac1*^+m/-^+ECL (white bar; total DAPI^+ ^nuclei counted in 27 fields; ONL: 26,175; INL: 11,968; GCL: 1,674). **(j) **Percentage of each retinal cell type based on total cell counts in wild-type (black bar; HC: 56 calbindin^+^/7,183 DAPI^+^; AC: 1,832 Pax6^+^/11,696 DAPI^+^; BP: 819 Chx10^+^/9,302 DAPI^+^; MG: 1,003 p27^+^/18,465 DAPI^+ ^nuclei; 537 CRALBP^+^/9,169 DAPI^+^), *Zac1*^+m/- ^without an ECL (grey bar; HC: 64 calbindin^+^/12,960 DAPI^+^; AC: 1,558 Pax6^+^/10,304 DAPI^+^; BP: 1,077 Chx10^+^/10,171 DAPI^+^; MG: 430 p27^+^/9,966 DAPI^+^; 332 CRALBP^+^/6,773 DAPI^+^) and *Zac1*^+m/-^+ECL retinae (white bar; HC: 11 calbindin^+^/1,924 DAPI^+^; AC: 2,068 Pax6^+^/11,302 DAPI^+^; BP: 646 Chx10^+^/9,157 DAPI^+^; MG: 395 p27^+^/9,921 DAPI^+^; 240 CRALBP^+^/3,319 DAPI^+^). AC, amacrine cell; BP, bipolar cell; HC, horizontal cell; MG, Müller glia.

By P3, 80% of Zac1+m/- pups die, with 50% dying within the first 24 hours [23]. To ensure we did not analyze surviving pups with unknown compensatory mechanisms, we studied only Zac1+m/- embryos collected prenatally, when Mendelian ratios of mutants were obtained. To circumvent the problem of retinogenesis not being complete until about P5–6 in the central retina [3], we cultured E18.5 retinae as explants for eight days in vitro (DIV), recapitulating the normal histogenic process [28]. Subsequent phenotypic analyses then focused on the central retina, adjacent to the transected optic nerve, where differentiation was complete. Strikingly, most Zac1+m/- explants (55%; n = 27/49) were thicker than their littermate controls, developing a distinct, ectopic cellular layer (ECL) between the INL and GCL (Figure 2b,d,f,f',h,h'; Additional data file 3). Wild-type (Figure 2a,c,e,e',g,g') and remaining Zac1+m/- retinae (not shown) acquired a normal trilaminar structure.

An ECL may develop due to an overall increase in retinal cell number and/or aberrant cellular migration. To determine if Zac1+m/- retinae were hypercellular, DAPI-labeled nuclei were counted. In ECL-containing Zac1+m/- explants (hereafter designated Zac1+m/-+ECL), there was a 1.34-fold increase in the number of INL cells (p < 0.001; 442.9 ± 17.9 cells/field; n = 9 retinae) compared to wild-type controls (329.5 ± 22.0 cells/field; n = 10) or non-ECL containing mutants (henceforth simply designated Zac1+m/-; 314.0 ± 22.1 cells/field; n = 3; Figure 2i). Strikingly, Zac1+m/-+ECL retinae also exhibited a 1.23-fold increase in ONL cells (p < 0.01; 969.4 ± 46.1 cells/field; n = 9) compared to wild-type controls (790.3 ± 40.7 cells/field; n = 10) or Zac1+m/- (735 ± 106.7 cells/field; n = 3; Figure 2i). In contrast, cellular contents of the GCL were comparable in wild-type (59.2 ± 3.1 cells/field; n = 10), Zac1+m/-+ECL (62.0 ± 4.3 cells/field; n = 9) and Zac1+m/- (55.3 ± 1.8 cells/field; n = 3) explants. Zac1 is, therefore, an essential negative regulator of retinal cell number and is also required to orchestrate appropriate cellular migration.

### The *Zac1*^+m/- ^ECL is composed of supernumerary amacrine cells

To identify the expanded cell population(s) in *Zac1*^+m/-^+ECL retinae, E18.5→8DIV explants were immunostained with cell type-specific markers. Strikingly, almost all cells in the *Zac1*^+m/- ^ECL expressed the homeodomain transcription factor Pax6 (Figure 2f,f'), which was also expressed by amacrine cells in the INL and GCL in wild-type (Figure 2e,e',f,f') and *Zac1*^+m/- ^(data not shown) E18.5→8DIV explants. Although Pax6 labels both amacrine cells and RGCs [29], RGCs rapidly undergo apoptosis following optic nerve transection (in explants [30]), allowing us to assign an amacrine cell identity to ECL cells. Accordingly, no other RGC markers (Brn3a/3b, Thy1.2; not shown) were detected in the ECL or GCL of wild-type or *Zac1*^+m/-^+ECL explants. Moreover, RGC differentiation is essentially complete by E18.5, and at this stage, equivalent numbers of RGCs were labeled by Brn3a (*p *= 0.95) and Brn3b (*p *= 0.23) in wild-type and *Zac1*^+m/- ^retinae, indicating *Zac1 *does not regulate RGC number (n = 3 for each; total n = 6; Additional data file 4). Furthermore, syntaxin, which labels amacrine cell membranes and processes in the inner plexiform layer (IPL; Figure 2g,g'), marked duplicated and disorganized synaptic plexi (IPL/IPL') in *Zac1*^+m/-^+ECL explants (Figure 2h,h'). Finally, amacrine cell subtype markers, including Bhlhb5, calbindin, GABA and the GlyT1 glycine transporter, were all expressed in *Zac1*^+m/- ^ECL (Additional data file 3 (k–p)).

Quantitation of Pax6^+ ^nuclei in E18.5→8DIV explants revealed a 1.31-fold increase (*p *< 0.01) in the percentage of amacrine cells in *Zac1*^+m/-^+ECL retinae, while *Zac1*^+m/-^explants contained wild-type proportions of these interneurons (wild type: 15.1 ± 0.5%; n = 4; *Zac1*^+m/-^: 15.2 ± 1.3%; n = 3; *Zac1*^+m/-^+ECL: 19.8 ± 1.3%; n = 3; Figure 2j). In contrast, all other INL cell types were present at equivalent ratios in wild-type and *Zac1*^+m/-^+/-ECL retinae, including bipolar cells (Chx10^+^; wild type: 8.7 ± 0.6%; n = 4; *Zac1*^+m/-^: 10.5 ± 0.9%; n = 3; *Zac1*^+m/-^+ECL: 7.2 ± 1.3%; n = 3), Müller glia (CRALBP^+^: wild type: 5.2 ± 0.3%; n = 4; *Zac1*^+m/-^: 4.3 ± 0.2%; n = 3; *Zac1*^+m/-^+ECL: 3.9 ± 0.5%; n = 3; p27^Kip1^+: wild type: 5.9 ± 0.5%; n = 4; *Zac1*^+m/-^: 5.1 ± 0.7%; n = 3; *Zac1*^+m/-^+ECL: 5.8 ± 0.1%; n = 3) and horizontal cells (calbindin^+^; identified also by morphology and apical location; wild type: 1.1 ± 0.3%; n = 3; *Zac1*^+m/-^: 0.8 ± 0.2%; n = 4; *Zac1*^+m/-^+ECL: 0.1 ± 0.1%; n = 3; Figure 2j; Additional data file 3 (m,n)).

Cones normally comprise only 3% of the murine photoreceptor pool [31]. In *Zac1*^+m/-^+ECL and wild-type retinae, similar numbers of cones were labeled with peanut agglutinin (PNA; *p *= 0.26; wild type: 50.4 ± 1.2cells/field; n = 3; *Zac1*^+m/-^+ECL: 64.6 ± 10.1cells/field; n = 3) and *s-opsin *(*p *= 0.70; wild type: 44.22 ± 8.91cells/field; n = 3; *Zac1*^+m/-^+ECL: 39.75 ± 6.66cells/field; n = 4; Additional data file 3 (g–j)). Instead, the vast majority of ONL cells in wild-type and *Zac1*^+m/-^+ECL explants expressed the rod-specific markers rhodopsin (Figure 2c, d) and Nr2e3 (not shown), indicating that the rod pool is expanded in *Zac1*^+m/-^+ECL retinae. *Zac1 *therefore ensures appropriate numbers of rod photoreceptors and amacrine cells are generated during development.

### Retinal progenitors divide ectopically in *Zac1 *mutants late in retinogenesis

**Figure 3:**
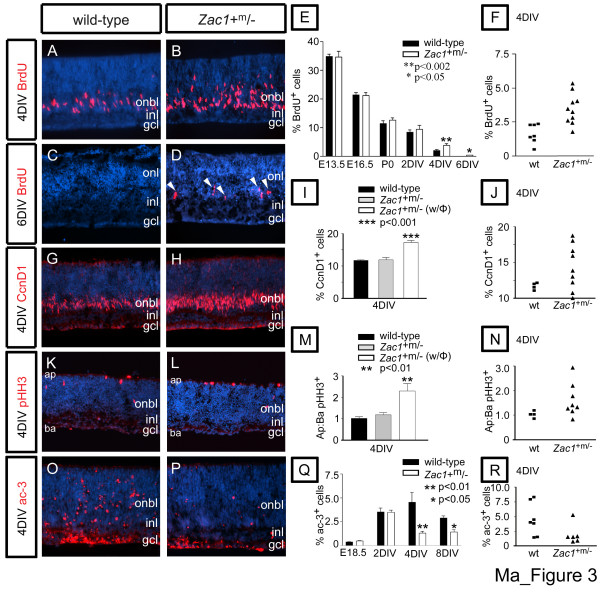
Loss of *Zac1 *results in increased proliferation and reduced apoptosis at a late stage of retinogenesis. **(a-d) **BrdU labeling (red) of E18.5 wild-type and *Zac1*^+m/- ^explants cultured 4DIV (a,b) or 6DIV (c,d). Arrowheads in (d) mark ectopic proliferating cells. **(e) **Percentage of BrdU^+ ^nuclei in wild-type (black bar; E13.5: 2,824 BrdU^+^/8,235 DAPI^+^; E16.5: 2,234 BrdU^+^/10,663 DAPI^+^; E18.5: 2,859 BrdU^+^/27,380 DAPI^+^; E18.5→2DIV: 4,371 BrdU^+^/54,554 DAPI^+^; E18.5→4DIV: 988 BrdU^+^/55,300 DAPI^+^; E18.5→6DIV: 0 in 9 fields) and *Zac1*^+m/- ^retinae (grey bars; E13.5: 3,555 BrdU^+^/10,413 DAPI^+^; E16.5: 3,369 BrdU^+^/15,707 DAPI^+^; E18.5: 2,212 BrdU^+^/17,642 DAPI^+^; E18.5→2DIV: 3,298 BrdU^+^/35,085 DAPI^+^; E18.5→4DIV: 3,474 BrdU^+^/97,499 DAPI^+^; E18.5→6DIV: 54 BrdU^+^/11,618 DAPI^+^). **(f) **BrdU-labeling indices of individual wild-type (squares) and *Zac1*^+m/- ^(triangles) E18.5→4DIV retinal explants. **(g,h) **E18.5→4DIV wild-type (g) and *Zac1*^+m/- ^(h) retinal explants labeled with CcnD1 (red). **(i) **Percentage of Ccdn1^+ ^cells in wild-type (black bar; 2,480 CcnD1^+^/21,329 DAPI^+^) and *Zac1*^+m/- ^without aberrant proliferation (grey bar; 3,156 CcnD1^+^/26,328 DAPI^+^) and with a proliferative phenotype (w/φ; white bar; 3,266 CcnD1^+^/18,709 DAPI^+^) at 4DIV. **(j) **Ccnd1-labeling indices of individual wild-type (squares) and *Zac1*^+m/- ^(triangles) E18.5→4DIV retinal explants. **(k,l) **E18.5→4DIV wild-type (k) and *Zac1*^+m/- ^(l) retinal explants labeled with pHH3 (red). **(m) **Apical (Ap) to basal (Ba) ratio of pHH3^+ ^cells in wild-type (black bar; 808 ap:791 ba pHH3^+^) and *Zac1*^+m/- ^without (grey bar; 971 ap:796 ba pHH3^+^) and with (w/φ; white bar; 1,012 ap:480 ba pHH3^+^) a proliferative phenotype at 4DIV. **(n) **Ap:Ba ratios of pHH3^+ ^cells in individual wild-type (squares) and *Zac1*^+m/- ^(triangles) E18.5→4DIV retinal explants. **(o-p) **Active caspase-3 (Ac-3) expression (red) in wild-type and *Zac1*^+m/- ^E18.5→4DIV explants. Blue is DAPI counterstain. **(q) **Percentage of apoptotic cells in the total population of wild-type (black bars; E18.5: 71 ac-3^+^/18,341 DAPI^+^; E18.5→2DIV: 532 ac-3^+^/14,995 DAPI^+^; E18.5→4DIV: 1,266 ac-3^+^/27,321 DAPI^+^; E18.5→8DIV: 294 ac-3^+^/10,209 DAPI^+^) and *Zac1*^+m/- ^(white bars; E18.5: 67 ac-3^+^/13,768 DAPI^+^; E18.5→2DIV: 457 ac-3^+^/13,195 DAPI^+^; E18.5→4DIV: 488 ac-3^+^/24,077 DAPI^+^; E18.5→8DIV: 212 ac-3^+^/14,377 DAPI^+^) retinae. **(r) **Distribution of individual wild-type (squares) and *Zac1*^+m/- ^(triangles) ac-3-labeling indices at 4DIV.

The hypercellularity of *Zac1*^+m/- ^retinae could arise due to additional rounds of cell division and/or a reduction in apoptosis. To determine if cell cycle exit was perturbed, S-phase progenitors were BrdU pulse-labeled 30 minutes prior to sacrifice. During embryogenesis (E13.5-E18.5) and in E18.5→2DIV explants, BrdU-labeling indices were similar in wild-type and Zac1+m/- retinae (Figure 3e). In contrast, in E18.5→4DIV Zac1+m/- explants, BrdU incorporation was elevated 2.1-fold over wild type (p < 0.002; Zac1+m/-: 3.4 ± 0.4%; n = 10; wild type: 1.6 ± 0.3%; n = 7; Figure 3a,b,e), although an ECL was not yet distinguishable. Notably, BrdU-labeling indices were variable in individual Zac1+m/- retinae, with about 50% of the mutant explants well above wild-type values (Figure 3f), a phenotypic distribution corresponding well with the proportion of mutant explants that later developed a hypercellular phenotype (see above). Furthermore, in 6DIV explants, when cell division had ceased in wild-type central retinae, BrdU uptake persisted in some mutants (p < 0.05; 0.5 ± 0.01%; n = 4/11; Figure 3c–e). As an independent cell cycle parameter, cyclin D1 (CcnD1) expressing cells were also elevated 1.48-fold (p < 0.001) over wild type (11.7 ± 0.2%; n = 4) in approximately half of the 4DIV Zac1+m/- explants (with phenotype: 17.3 ± 0.6%; n = 4/9; without phenotype: 12.0 ± 0.7%; n = 5/9; Figure 3g–j). Cell proliferation was thus specifically elevated at late stages of retinogenesis in *Zac1 *mutants.

**Figure 4:**
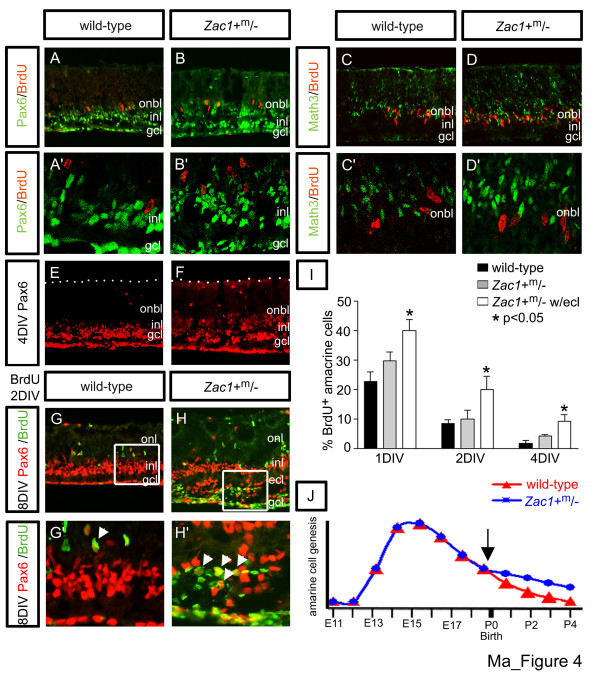
Amacrine cell genesis is elevated postnatally in *Zac1*^+m/- ^retinae. **(a-d) **E18.5→4DIV wild-type (a,a',c,c') and *Zac1*^+m/- ^(b,b',d,d') explants co-labeled with BrdU (red, S-phase) and Pax6 (green; amacrine cells (a,a',b,b') or Math3 (green; amacrine and bipolar precursors in INL (c,c',d,d'). **(e,f) **E18.5→4DIV explants labeled with Pax6 alone (red). **(g,g',h,h') **Birthdating of E18.5→8DIV wild-type (g,g') and *Zac1*^+m/- ^(h,h') retinal explants exposed to BrdU (green) at 2DIV and co-labeled with anti-Pax6 (red). BrdU/Pax6 double^+ ^cells are marked by arrowheads in (g',h'), which are high magnification images of boxed areas in (g,h), respectively. **(i) **Percentage of BrdU/Pax6 double^+ ^nuclei (amacrine cells born at time of BrdU exposure) in wild-type (black bar; BrdU at 1DIV: 562 BrdU/Pax6 double^+^/2,385 Pax6^+^; 2DIV: 527 BrdU/Pax6 double^+^/6,022 Pax6^+^; 4DIV: 77 BrdU/Pax6 double^+^/1,496 Pax6^+^; all counts in 8DIV explants) and *Zac1*^+m/- ^explants without an ECL (grey bar; BrdU at 1DIV: 1,307 BrdU/Pax6 double^+^/4,084 Pax6^+^; 2DIV: 527 BrdU/Pax6 double^+^/4,926 Pax6^+^; 4DIV: 75 BrdU/Pax6 double^+^/1,660 Pax6^+^) and *Zac1*^+m/-^+ECL explants (white bar; BrdU at 1DIV: 2,126 BrdU/Pax6 double^+^/6,107 Pax6^+^; 2DIV: 883 BrdU/Pax6 double^+^/4,386 Pax6^+^; 4DIV: 335 BrdU/Pax6 double^+^/3,587 Pax6^+^). **(j) **Model of amacrine cell genesis in wild-type (red line) versus *Zac1*^+m/-^+ECL (blue line) retinae.

Ectopic division could occur if progenitors cycled more extensively and/or committed precursors failed to exit the cell cycle. Retinal progenitors are defined by cell cycle-dependent, interkinetic nuclear movements, with G2/M-phase, phospho-histoneH3 (pHH3)-expressing nuclei lining the apical surface (Figure 3k,l), while S-phase nuclei lie more basal in the onbl [32] (Additional data file 1 (e)). This contrasts to committed precursors that migrate towards the vitreal (basal) surface of the inbl to initiate formation of the mature retinal layers. We thus used mitotic position to distinguish proliferating progenitors (apical mitoses) versus precursors (basal mitoses) [33]. In *Zac1*^+m/- ^retinae, the proportion of pHH3-labeled nuclei was biased towards apical compartments in many *Zac1*^+m/- ^4DIV explants (apical to basal ratio: wild type: 1.02 ± 0.07; n = 10; *Zac1*^+m/-^+phenotype: 2.30 ± 0.35; n = 3/8; *Zac1*^+m/-^: 1.19 ± 0.10; n = 5/8; Figure 3k–e), consistent with an increase in progenitor and not precursor cell divisions. Accordingly, most Pax6^+ ^amacrine precursors did not incorporate BrdU after a 30 minute exposure in wild-type or *Zac1*^+m/- ^4DIV explants (Figure 4a,a',b,b'; Additional data file 5 (j)). Similarly, double labeling with Math3, an amacrine and bipolar precursor marker, revealed very few Math3/BrdU double^+ ^cells in wild-type and *Zac1*^+m/- ^explants (Figure 4c,c',d,d'). Therefore, retinal progenitor cells and not committed precursors are dependent on *Zac1 *for cell cycle exit.

### Apoptosis is reduced during late developmental stages in *Zac1*-deficient retinae

Compensatory mechanisms exist in the retina to ensure that cellular content remains constant, with excess proliferation often balanced by an increase in apoptosis [34,35]. Given that *Zac1 *induces apoptosis when misexpressed in cell lines [24], we tested if it were also required for the normal program of cell death in the retina, using activated-caspase-3 (ac-3), a downstream effector and early marker of commitment to the cell death pathway [36]. During embryonic retinal development, apoptosis peaks during the optic cup stage (E10–E11) in the presumptive retinal pigmented epithelium (rpe) and optic stalk and again between E15.5–E17.5, primarily in retinal cells adjacent to the optic nerve head [37-40]. We analyzed ac-3 staining in wild-type (n = 6) and *Zac1 *mutant retinae (n = 6) at E10.5 and E15.5 but did not observe more than a few apoptotic cells per retinal section in either genotype (Additional data file 5 (a-d)). Similarly, at E18.5 (*p *= 0.14; wild type: 0.4 ± 0.02%; n = 3; *Zac1*^+m/-^: 0.5 ± 0.03%; n = 3) and in E18.5→2DIV explants (*p *= 0.93; wild type: 3.5 ± 0.4%; n = 3; *Zac1*^+m/-^: 3.5 ± 0.2%; n = 3), comparable levels of apoptosis were observed in both genotypes (Figure 3q). In contrast, after 4 and 8DIV, there were 3.48-fold (*p *< 0.01; wild type: 4.5 ± 1.0%; n = 7; *Zac1*^+m/-^: 1.3 ± .0.2%; n = 5/6) and 2.02-fold (*p *< 0.05; wild type: 2.9 ± 0.2%; n = 3; *Zac1*^+m/-^: 1.4 ± 0.3%; n = 4) reductions, respectively, in the number of ac-3^+ ^retinal cells in *Zac1*^+m/- ^explants (Figure 3o–r).

The reduction in cell death in *Zac1*^+m/- ^explants could contribute to the increase in amacrine and rod cell numbers. However, the number of ac-3/Pax6-double^+ ^amacrine cells was similar in E18.5→4DIV explants from both genotypes (*p *= 0.15; wild type: 1.6 ± 0.4%; n = 3; *Zac1*^+m/-^: 0.9 ± 0.1%; n = 3; Additional data file 5 (e–i)). In contrast, there was a 1.82-fold reduction in ac-3^+ ^ONL photoreceptors in *Zac1*^+m/- ^E18.5→8DIV explants (*p *< 0.05; wild type: 2.9 ± 0.2%; n = 3; *Zac1*^+m/-^: 1.60 ± 0.4%; n = 4). *Zac1 *deficiency therefore perturbs pro-apoptotic pathways that adjust cell numbers at late stages of retinogenesis, likely contributing to the increase in rod cell number.

### *Zac1 *is a direct negative regulator of proliferation and rod differentiation

**Figure 5:**
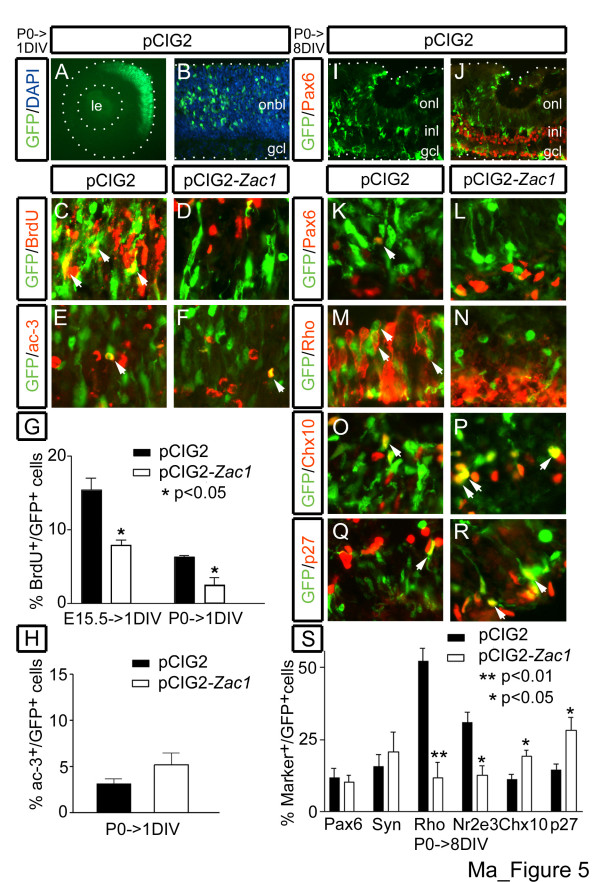
*Zac1 *inhibits cell division and rod fate specification. **(a-f) **P0 retinae electroporated with pCIG2 control (a-c,e) or pCIG2-*Zac1 *(d,f) cultured 1DIV. GFP^+ ^electroporated cells (green (a-f)) labeled with anti-BrdU (red (c,d)) and anti-ac-3 (red (e,f)). Blue in (b) is DAPI counterstain. **(g) **Percentage of GFP^+ ^cells that incorporated BrdU after electroporation of pCIG2 (black bar; E15.5→1DIV: 88 BrdU/GFP double^+^/542 GFP^+^; P0→1DIV: 124 BrdU/GFP double^+^/1,784 GFP^+^) and pCIG2-*Zac1 *(white bar; E15.5→1DIV: 24 BrdU/GFP double^+^/290 GFP^+^; P0→1DIV: 14 BrdU/GFP double^+^/816 GFP^+^). **(h) **Percentage of GFP^+ ^cells that expressed ac-3 in P0→1DIV retinae electroporated with pCIG2 (black bar; 157 ac-3/GFP double^+^/5,402 GFP^+^) and pCIG2-*Zac1 *(white bar; 97 ac-3/GFP double^+^/1,808 GFP^+^). **(i-r) **P0→8DIV retinae electroporated with pCIG2 (i-k,m,o,q) or pCIG2-*Zac1 *(l,n,p,r). GFP^+ ^electorporated cells (green (i-r)) co-labeled with Pax6 (red; amacrine cells (j-l)), rhodopsin (red; rods (m,n)), Chx10 (red; bipolar (o,p)) and p27^Kip1 ^(red; Müller glia (q,r)). **(s) **Percentage of GFP^+ ^cells expressing cell-type specific markers post-electroporation of pCIG2 (black bar; 290 Pax6/GFP double^+^/3,939 GFP^+^; 81 syntaxin/GFP double^+^/552 GFP^+^; 955 rhodopsin/GFP double^+^/1,751 GFP^+^; 384 Nr2e3/GFP double^+^/1,261 GFP^+^; 279 Chx10/GFP double^+^/3,146 GFP^+^; 520 p27/GFP double^+^/3,846 GFP^+^) or pCIG2-*Zac1 *(white bar; 140 Pax6/GFP double^+^/1,284 GFP^+^; 83 syntaxin/GFP double^+^/376 GFP^+^; 56 rhodopsin/GFP double^+^/356 GFP^+^; 131 Nr2e3/GFP double^+^/816 GFP^+^; 263 Chx10/GFP double^+^/1,455 GFP^+^; 541 p27/GFP double^+^/1,888 GFP^+^). Arrowheads mark double^+ ^cells. le, lens; Rho, Rhodopsin; Syn, Syntaxin.

To test if *Zac1 *was a direct negative regulator of amacrine and rod cell fates, we established a gain-of-function assay, electroporating retinal explants with a pCIG2 vector, containing an internal ribosome entry site (IRES) 2-enhanced green fluorescent protein (EGFP) cassette, or a pCIG2-*Zac1 *vector, expressing both EGFP and *Zac1 *(Figure 5). E15.5 and P0 retinal explants misexpressing *Zac1 *were BrdU-pulse labeled 24 hours post-electroporation, revealing 1.96-fold and 2.49-fold reductions, respectively, in the number of BrdU/EGFP-double^+ ^cells compared to controls at E15.5 (*p *< 0.05; pCIG2: 15.4 ± 1.6%; n = 3; *Zac1*: 7.9 ± 0.8%; n = 3; Figure 5g) and P0 (*p *< 0.05; pCIG2: 6.3 ± 0.3%; n = 3; *Zac1*: 2.5 ± 1.0%; n = 3; Figure 5a–d,g). *Zac1 *therefore promotes cell cycle exit and/or increases cell cycle length in the murine retina. In contrast, *Zac1 *misexpression did not increase the number of ac-3^+ ^cells compared to controls 24 hour post-electroporation at P0 (*p *= 0.2; pCIG2: 3.1 ± 0.6%; n = 3; *Zac1*: 5.2 ± 1.3%; n = 3; Figure 5e,f,h), indicating that *Zac1 *is not sufficient to induce retinal apoptosis.

To determine if *Zac1 *was a direct, negative regulator of rod and/or amacrine fates, we examined the molecular phenotype of retinal cells electroporated at P0 and cultured 8DIV. No differences were observed in the ratio of GFP^+ ^cells that became Pax6^+ ^amacrine cells after electroporation of pCIG2 (*p *= 0.73; 11.7 ± 3.4%; n = 6; Figure 5i–,k,s) versus pCIG2-*Zac1 *(10.2 ± 2.3%; n = 6; Figure 5l,s). Similarly, misexpression of *Zac1 *at E15.5 and E17.5, during the peak of amacrine cell genesis, did not affect amacrine cell number (Additional data file 6). In contrast, *Zac1 *misexpression at P0 resulted in a 4.49-fold reduction in rhodopsin^+ ^rods (*p *< 0.01; pCIG2: 52.3 ± 4.5%; n = 3; *Zac1*: 11.6 ± 5.4%; n = 3; Figure 5m,n,s) and a 2.43-fold reduction in Nr2e3-labeled rods (*p *< 0.05; pCIG2: 30.8 ± 3.6%; n = 3; *Zac1*: 12.7 ± 3.1%; n = 3; Figure 5s). *Zac1*-misexpressing progenitors instead preferentially differentiated into Chx10^+ ^bipolar cells (1.72-fold increase; *p *< 0.05; pCIG2: 11.1 ± 1.8%; n = 6; *Zac1*: 19.1 ± 2.2%; n = 6) and p27^Kip1+ ^Müller glia (1.95-fold increase; *p *< 0.05; pCIG2: 14.4 ± 2.2%; n = 6; *Zac1*: 28.1 ± 4.5%; n = 6), cells types normally generated along with rods postnatally (Figure 5o–s). *Zac1 *is thus a potent inhibitor of a rod fate but does not directly suppress amacrine cell genesis.

### Elevated amacrine cell genesis continues for a prolonged period in *Zac1 *mutants

To understand how *Zac1 *controls amacrine cell numbers, we next determined when ectopic amacrine cells first appeared in *Zac1*^+m/- ^retinae. In mouse, amacrine cell genesis normally peaks at E15.5, tapering off before birth [3] (Figure 4j). At E18.5, genes involved in amacrine fate specification/differentiation, including *Math3*, *Foxn4*, *NeuroD*, *Pax6 *and *Barhl2 *[41-43], were expressed in an indistinguishable manner in wild-type and *Zac1*^+m/- ^retinae, as were several other genes involved in the specification of all other cell types (Additional data file 7). Cell fate specification was thus grossly normal in E18.5 *Zac1*^+m/- ^retinae. In contrast, in E18.5→4DIV *Zac1*^+m/- ^explants, Pax6 (Figure 4a,a',b,b',e,f), *Six3*, *Barhl2 *and *Math3 *(Additional data file 8 (i–n)) expression increased, suggesting the amacrine cell population expanded during early postnatal stages in *Zac1*^+m/- ^retinae.

To verify that amacrine genesis increased postnatally in *Zac1*^+m/- ^retinae, we performed birthdating. E18.5 retinal explants were labeled with BrdU after 1, 2 and 4DIV and then cultivated for 8DIV (Figure 4g,g',h,h'). More BrdU^+^/Pax6^+ ^amacrine cells were born at 1DIV (1.76-fold increase; *p *< 0.05; wild type: 22.7 ± 3.4%; n = 3; *Zac1*^+m/-^+ECL: 39.9 ± 3.8%; n = 4; *Zac1*^+m/-^: 29.8 ± 3.0%; n = 3), 2DIV (2.34-fold increase; *p *< 0.05; wild type: 8.6 ± 1.3%; n = 7; *Zac1*^+m/-^+ECL: 20.0 ± 4.4%; n = 4; *Zac1*^+m/-^: 10.1 ± 2.8%; n = 6) and 4DIV (5.42-fold increase; *p *< 0.05; wild type: 1.7 ± 1.0%; n = 6; *Zac1*^+m/-^+ECL: 9.2 ± 2.4%; n = 3; *Zac1*^+m/-^: 4.3 ± 0.4%; n = 2; Figure 4g,g',h,h',i) in *Zac1*^+m/-^+ECL explants compared to wild type, confirming that the period of amacrine cell genesis was prolonged.

### Negative feedback signals are deficient in *Zac1*^+m/- ^amacrine cells

**Figure 6:**
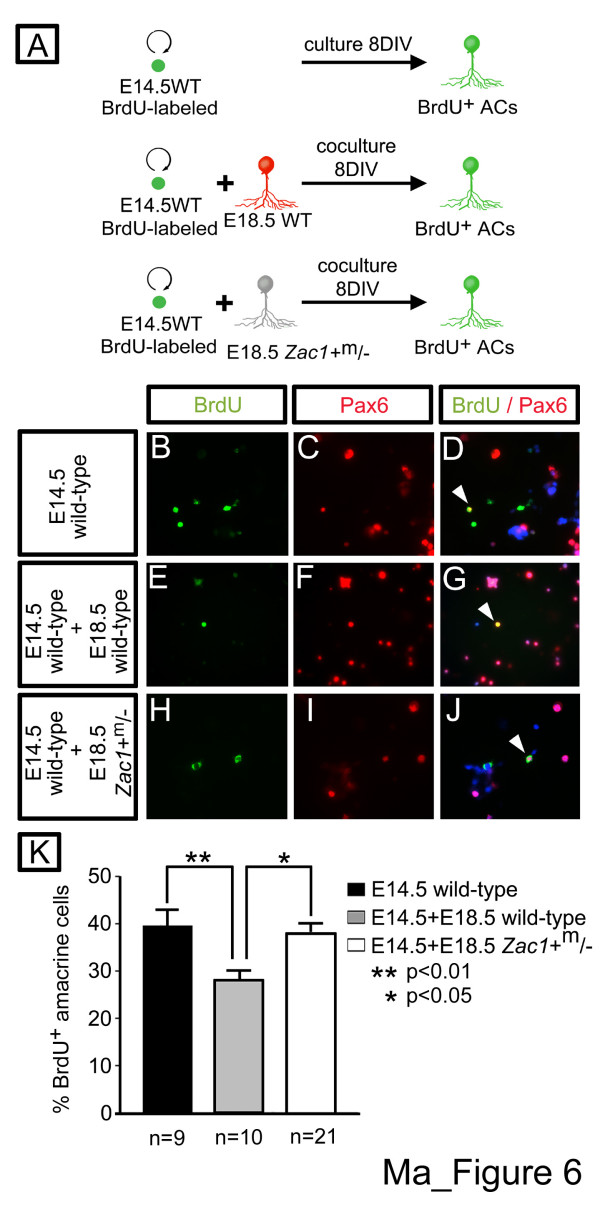
*Zac1*^+m/- ^retinae lose amacrine cell feedback inhibition. **(a) **Schematic of aggregation assay protocol. **(b-j) **Immunolabeling of dissociated cell pellets with Pax6 (red (c,d,f,g,i,j)), BrdU (green (b,d,e,g,h,j)) and merged image with DAPI (blue (d,g,j)). E14.5 progenitors cultured alone (b-d) or with E18.5 wild-type (e-g) or *Zac1*^+m/- ^(h-j) retinal cells. Arrowheads mark Pax6/BrdU double^+ ^nuclei (d,g,j). **(k) **Percentage of BrdU^+ ^E14.5 cells that differentiated into Pax6^+ ^amacrine cells when cultured alone (black bar; 1,085 BrdU/Pax6 double^+^/2,892 BrdU^+^) or with E18.5 wild-type (grey bar; 853 BrdU/Pax6 double^+^/3,215 BrdU^+^) or *Zac1*^+m/- ^(white bar; 2,559 BrdU/Pax6 double^+^/7,196 BrdU^+^) retinal cells. n indicates number of individual retinal aggregates quantified.

Our data suggested that the 'stop' or negative feedback signals that normally limit amacrine cell production later in development [13,14] were deficient in *Zac1*^+m/- ^retinae (Figure 4j). To thus test if *Zac1 *was an essential component of the amacrine cell negative feedback loop, we performed aggregation assays. Dissociated E14.5 wild-type retinal cells pre-labeled with BrdU were either cultured alone as intact pellets or in pellet aggregations with a 20-fold excess of dissociated E18.5 wild-type or *Zac1*^+m/- ^retinal cells, the latter populations serving as a source of amacrine cell feedback signals (Figure 6a). After 8DIV, pellets were dissociated and Pax6^+^/BrdU^+ ^amacrine cells derived from E14.5 progenitors were quantified (Figure 6b–J). Of the E14.5 cells cultured alone, 39.6 ± 3.4% (n = 9; 3 independent experiments) of BrdU^+^cells became Pax6^+ ^amacrine cells (Figure 6b–d,k). Consistent with feedback signals being emitted from differentiating, E18.5 wild-type cells, in co-cultures, amacrine cell development from the E14.5-labeled cohort was reduced 1.40-fold (*p *< 0.01; 27.9 ± 2.0%; n = 10; Figure 6e–g,k). In contrast, amacrine cell development from the E14.5 cohort was restored to normal levels (compared to E14.5 cells alone) in mixed aggregates containing E18.5 *Zac1*^+m/- ^cells (*p *< 0.05, 37.7 ± 2.4%; n = 21), indicative of impaired negative feedback (Figure 6h–k). *Zac1 *is thus required in postnatal retinal cells to negatively regulate amacrine cell genesis.

### TGFβ signaling inhibits amacrine cell genesis in the retina

**Figure 7:**
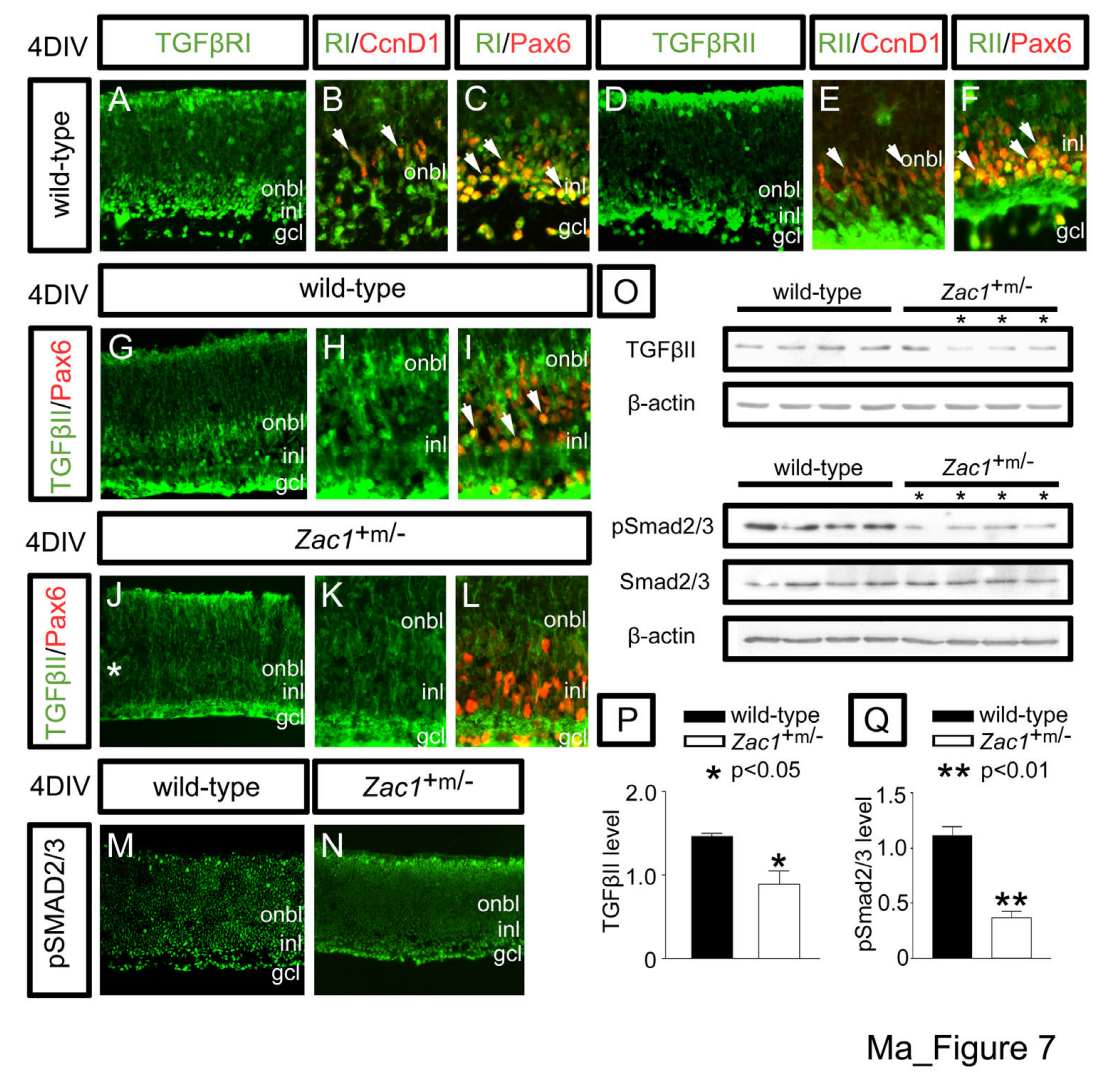
*Zac1 *regulates TGFβII signaling in the retina. **(a-f) **Co-expression of TGFβRI (green (a-c)) and TGFβRII (green (d-f)) with Ccnd1 (red, proliferating progenitors (b,e)) and Pax6 (red, amacrine cells (c,f)) in E18.5 > 4DIV wild-type retinal explants. **(g-l) **TGFβII expression in E18.5→4DIV wild-type (green (g-i)) and *Zac1*^+m/- ^(green (j-l)) retinal explants co-labeled with Pax6 (red, amacrine cells (i,l)). Arrowheads mark double^+ ^cells. Asterisk in (j) marks reduction in onbl/INL expression. **(m,n) **Expression of pSmad2/3 in E18.5→4DIV wild-type (m) and *Zac1*^+m/- ^(n) retinal explants. **(o) **Western blot analysis of TGFβII, pSmad2/3, total Smad2/3, and β-actin. Asterisks in (o) indicate mutants with reduced expression of TGFβII or pSmad2/3. **(p,q) **Quantitation of expression levels normalized to β-actin via densitometry for TGFβII (p) and pSmad2/3 (q).

The cell non-autonomous requirement for *Zac1 *as a negative regulator of amacrine cell production implied that this transcription factor must regulate the expression of an unknown secreted signal. We focused on TGFβ cytokines, given their role in feedback control in other systems. Specifically, we studied TGFβII, which regulates cell cycle exit at late stages of rat retinogenesis [44], corresponding to the period when *Zac1*^+m/- ^cells proliferated aberrantly. In E18.5 > 4DIV explants, the cognate receptors, TGFβRI and TGFβRII, were expressed at low levels in dividing, Ccnd1^+ ^progenitors (Figure 7a,b,d,e) and at higher levels in Pax6^+ ^amacrine cells (Figure 7c,f). TGFβII was similarly expressed in Pax6^+ ^amacrine cells in the GCL and INL (Figure 7g–i) and at lower levels in Ccnd1^+ ^progenitors in the onbl (not shown). Thus, TGFβII signaling could correspond to the amacrine cell stop signal.

In *Zac1 *mutants, a notable reduction in TGFβII expression was observed in onbl progenitors and in Pax6^+ ^amacrine cells in the INL, while GCL levels were similar to wild type (Figure 7j–l). An overall reduction in TGFβII levels was confirmed by western blot, demonstrating that the 25 kDa isoform (note: labile 12 kDa mature form not detected) was reduced in most (n = 8/12) *Zac1*^+m/- ^explants (*p *< 0.05; signal normalized to β-actin; wild type: 1.5 ± 0.04; n = 4; *Zac1*^+m/-^: 0.9 ± 0.2; n = 3/4; Figure 7o,p). To confirm that TGFβ signaling was indeed reduced in *Zac1*^+m/- ^retinae, we examined expression of the downstream effector, pSmad 2/3. In E18.5→4 DIV wild-type explants, pSmad2/3 was expressed at diffuse levels throughout the retinae, but at significantly higher levels in the GCL and the basal half of the INL, where differentiated amacrine cells reside, as well as at lower levels in dividing progenitor cells in the onbl (Figure 7m). In contrast, pSmad2/3 levels were decreased in the INL and onbl progenitors in *Zac1*^+m/- ^explants (Figure 7n). Accordingly, western blot analysis revealed a significant reduction in pSmad2/3 protein levels in *Zac1*^+m/- ^versus wild-type E18.5 > 4 DIV explants when normalized to β-actin (*p *< 0.01; n = 6/8 mutants analyzed), while total Smad2/3 protein levels were similar in both genotypes (n = 4 for each genotype; Figure 7o,q). TGFβ signaling was thus attenuated in *Zac1*^+m/-^retinae.

**Figure 8:**
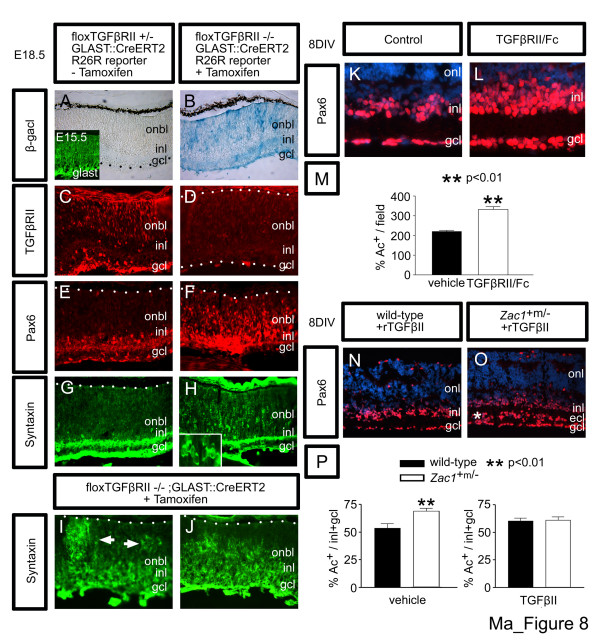
TGFβII negatively regulates amacrine cell genesis. **(a,b) **X-gal staining of E18.5 GLAST::CreERT2^+/-^;R26R reporter^+ ^transgenic without (a) and with (b) administration of tamoxifen at E16. Inset in (a) shows GLAST immunostaining of E15.5 retina. **(c-j) **Analysis of TGFβRII^+/-^;GLAST::CreERT2^+/-^;R26R^+ ^(c,e,g) and TGFβRII^-/-^;GLAST::CreERT2^+/-^;R26R^+ ^(d,f,h,i,j) retinae immunostained with anti-TGFβRII (c,d), Pax6 (e,f) and syntaxin (g-j). Arrowheads in (i) mark ectopic amacrine cell clusters. TGFβRII^-/- ^retinae in panels (d-h) and (i,j) are from two different mutant embryos. **(k,l) **E18.5 > 8DIV retinal explants cultured with vehicle control (k) or TGFβRII-Fc (l) and labeled with anti-Pax6. **(m) **Percentage of Pax6^+ ^amacrine cells/field in vehicle control (black bar; 2,148 Pax6^+ ^in 10 fields) and TGFβRII-Fc treated (white bar; 4,966 Pax6^+ ^in 15 fields) retinal explants. **(n,o) **E18.5→8DIV wild-type (n) or *Zac1*^+m/- ^(o) retinal explants cultured with rTGFβII. Asterisk in (o) indicates ECL formation in *Zac1*^+m/- ^retinae even in the presence of rTGFβII. **(p) **Percentage of amacrine cells in wild-type explants (black bar; vehicle control: 1,761 Pax6^+^/3,353 DAPI^+^; rTGFβII: 2,605 Pax6^+^/4,301 DAPI^+ ^in INL+GCL) and *Zac1*^+m/-^+ECL explants (white bar; vehicle control: 2,232 Pax6^+^/3,328 DAPI^+^; rTGFβII: 3,243 Pax6^+^/5,282 DAPI^+ ^in INL+GCL).

To determine if reduced TGFβ signaling results in amacrine cell expansion, conditional TGFβRII mutants were analyzed. Mice heterozygous (Figure 8a,c,e,g) or homozygous (Figure 8b,d,f,h,i,j) for a floxed mutant allele of TGFβRII (hereafter referred to as flTGFβRII; [45]) were crossed with mice carrying a R26R reporter and a GLAST::CreERT2 knock-in allele [46]. GLAST was expressed in the embryonic retina (Figure 8a, inset) and, accordingly, tamoxifen administered at E16 specifically induced CreERT2 recombinase activity in the E18.5 retina as evidenced by R26R reporter expression (that is, X-Gal staining in tamoxifen injected (Figure 8b) and not un-injected control (Figure 8a) retinae). Accordingly, expression of TGFβRII was reduced in E18.5 flTGFβRII^-/- ^(Figure 8d) compared to flTGFβRII^+/- ^retinae (Figure 8c). An overt expansion of the amacrine cell layer, as labeled by Pax6 (Figure 8e,f) and syntaxin (Figure 8g–j), was also evident in tamoxifen-induced E18.5 flTGFβRII^-/- ^mutant retinae (Figure 8f,h and Figure 8i,j show different mutants) compared to wild-type controls (Figure 8e,g).

While the analysis of TGFβRII mutants supported a role for this signaling pathway in regulating amacrine cell number, we were precluded from analyzing the effects of mutating TGFβRII at postnatal stages as the mutants unexpectedly died at early postnatal stages. We therefore used a complementary pharmacological approach to mimic the late reduction in TGFβ signaling observed in *Zac1 *mutant retinae. The pharmacological inhibition of TGFβII in the early postnatal rat retina increases proliferation and cell number [44], but specific effects on amacrine cell genesis were not analyzed. In accordance with experiments in rat [44], addition of 0.5 μg/ml soluble TGFβRII-Fc receptor to E18.5→8DIV retinal explants resulted in a 1.55-fold increase in INL/GCL cell number compared to vehicle controls (*p *< 0.01; control: 387.1 ± 35.0 cells/field; n = 3; TGFβRII-Fc: 601.6 ± 62.1 cells/field; n = 3; Figure 8k,l), while 0.1 μg/ml had no effect (not shown). Moreover, the inhibition of TGFβII signaling resulted in a 1.50-fold increase in the absolute number of amacrine cells (*p *< 0.01; vehicle control: 220.2 ± 5.9 cells/field; n = 3; TGFβRII-Fc: 331.2 ± 15.3 cells/field; n = 3; Figure 8k–m). These results are consistent with a requirement for TGFβ signaling to negatively regulate amacrine cell number during development.

Next, to show that attenuation of TGFβ signaling underlies amacrine cell expansion in *Zac1*^+m/- ^retinae, we performed a rescue experiment. Recombinant TGFβII (or vehicle control) was added to wild-type and *Zac1*^+m/- ^E18.5→8DIV explants. In control explants, the percentage of Pax6^+ ^amacrine cells was elevated 1.38-fold in *Zac1*^+m/-^+ECL versus wild-type explants (*p *< 0.01; wild type: 53.6 ± 4.2% INL/GCL cells; n = 4; *Zac1*^+m/-^+ECL; 68.7 ± 4.8% INL/ECL/GCL cells; n = 3; Figure 8p). In contrast, following exposure to TGFβII for 8DIV, the percentage of amacrine cells was equivalent in wild-type and *Zac1*^+m/-^+ECL explants (wild type: 60.4 ± 2.5% INL/GCL cells; n = 3; *Zac1*^+m/-^+ECL: 61.5 ± 2.9% INL/ECL/GCL cells; n = 3; Figure 8n–p). Strikingly, however, an ECL still formed in TGFβII-treated *Zac1*^+m/- ^explants (Figure 8o), suggesting that an alternative, non-TGFβ-mediated pathway underlies amacrine cell migration defects. This is also consistent with the inability of TGFβRII-Fc to induce an ECL (Figure 8l). These studies implicate attenuated TGFβII signaling in amacrine cell expansion in *Zac1*^+m/- ^retinae.

## Discussion

**Figure 9:**
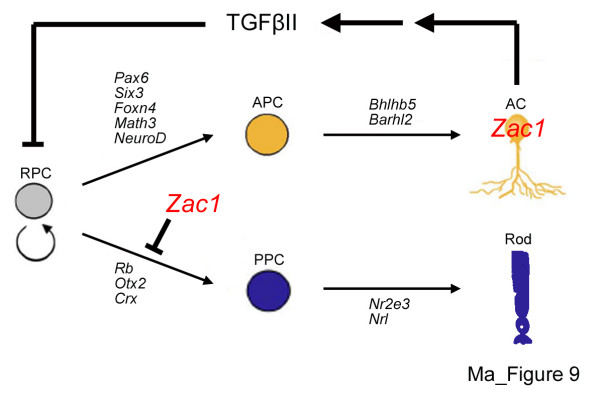
Model of *Zac1 *function in the retina. *Zac1 *negatively regulates amacrine cell number cell non-autonomously, controlling TGFβII expression, which inhibits amacrine cell genesis at threshold levels (negative feedback). In contrast, *Zac1 *negatively regulates rod number cell-autonomously. *Zac1 *negatively regulates (either directly or indirectly) the expression of genes involved in the specification/differentiation of an amacrine cell (*Pax6*, *Six3*, *Foxn4*, *Math3*, *NeuroD*, *Bhlhb5*, *Barhl2*) and rod cell (*Rb*, *Otx2*, *Crx*, *Nr2e3*, *Nrl*) fate by controlling the decision by retinal progenitor cells to differentiate along these lineages. AC, amacrine cell; APC, amacrine precursor cell; PPC, photoreceptor precursor cell; RPC, retinal progenitor cell.

The development of a functional retina requires that appropriate numbers of each cell type be generated. Hence, the molecular events that guide cell fate specification and differentiation must be tightly coordinated with those that govern cell number control. Here we demonstrate that the *Zac1 *tumor suppressor is an essential negative regulator of retinal size, controlling the absolute number of rod photoreceptors and amacrine cells generated during development. Strikingly, *Zac1 *regulates rod and amacrine cell genesis through distinct cell autonomous and non autonomous mechanisms, respectively (Figure 9). While *Zac1 *is a direct negative regulator of a rod photoreceptor fate, it regulates amacrine cell genesis by controlling the expression of TGFβII, which serves as an amacrine cell negative feedback signal. *Zac1 *and *TGFβII *thus join a growing list of tumor suppressor genes with established roles in retinogenesis (for example, *Rb*, *p53*, *p27*^Kip1 ^[33,35,40,44,47-52]), but are the first tumor surveillance molecules shown to control neuronal number through a negative feedback or 'cell sensing' mechanism.

### *Zac1 *promotes cell cycle exit and apoptosis in the developing retina

The widespread expression of *Zac1 *in dividing progenitor cells in the retina (this study) and throughout the developing neural tube [25,53-55] suggested that it would have an early role in neural development. Unexpectedly, we found that in the murine retina, *Zac1 *function is restricted to the early postnatal period. While we cannot rule out the possibility that *Zac1 *functions redundantly with other factors to regulate early events in retinal development, we would predict that the tumor suppressor-like properties of *Zac1 *would have to be actively suppressed during early retinal development as most cells that express *Zac1 *at these stages continue to divide for some time. Indeed, we show here that *Zac1 *is required to promote cell cycle exit only at late stages of retinogenesis, a context dependency that is also characteristic of other tumor suppressor genes and oncogenes [56]. Specifically, we show that, in *Zac1 *mutants, retinal progenitor cells divide excessively, similar to *p27*^Kip1 ^mutants [35,52] and in contrast to *Rb *mutants, where committed precursors instead fail to exit the cell cycle [33,47,48]. Our demonstration that cyclin D1 expression is upregulated in *Zac1*^+m/- ^retinae provides some insight into the molecular mechanisms underlying *Zac1*-mediated control of the cell cycle. However, several observations make it unlikely that *Zac1 *functions directly through p27^Kip1 ^or the related cyclin dependent kinase (CDK) inhibitor (CDKI) p57^Kip2 ^to regulate cell cycle exit. Firstly, p27^Kip1 ^is not required in a temporally restricted manner in the retina, and p57^Kip2 ^is only required at early stages of retinal development [35,52,57], which contrasts with the late temporal requirement for *Zac1*. Furthermore, expression of the Kip family CDKIs was not altered in *Zac1 *mutants, and while there was an increase in p27^Kip1 ^expression following *Zac1 *misexpression, it was specific to Müller glia, where this CDKI is normally expressed, and not observed in other cell types. Moreover, a previous cell culture study reported that *Zac1 *promoted cell cycle exit independently of Kip-family CDKIs or other classic cell cycle regulators such as Rb [24].

### *Zac1 *functions as a direct negative regulator of rod cell fate

The requirement for *Zac1 *to promote cell cycle exit and apoptosis at late stages of retinal development likely contributes to the formation of hypercellular retinae in mutants, but does not explain why rod photoreceptors and amacrine cells are the only two cell types that are specifically expanded. Strikingly, misexpression of *Zac1 *robustly inhibited rod differentiation, implicating *Zac1 *as a *bona fide *negative regulator of this cell fate. Accordingly, *Zac1 *expression declines in progenitor cells at P0 when rod photoreceptor genesis begins to peak. *Zac1 *is also not expressed in differentiated ONL photoreceptors. However, we cannot rule out the possibility that cell non-autonomous mechanisms may also underlie the expansion of the rod pool in *Zac1*^+m/- ^retinae. Indeed, we found that the generation of excess rods is directly linked to the formation of an ECL, both occuring in the same approximately 55% of *Zac1*^+m/- ^retinae. Notably, we implicated attenuated TGFβ signaling [58], a proapoptotic pathway, in the amacrine cell expansion. However, reduced TGFβ signaling may also underlie the decreased apoptosis we observed in *Zac1*^+m/- ^ONLs, consequently contributing to the expansion of the rod pool.

*Zac1 *misexpression also increased bipolar and Müller glial production in our gain-of-function assays, but rather than proposing that *Zac1 *is instructive for these fates, we favor the interpretation that progenitor cells prevented from adopting a rod fate instead acquire later-born fates by default. Accordingly, in *Zac1*^+m/- ^retinae, we did not observe compensatory decreases in bipolar and Müller glial cell number. Nevertheless, in *Xenopus*, murine *Zac1 *also promoted Müller glial as well as RGC genesis, suggesting it might be instructive for a glial identity in different vertebrate species [26]. However, there are numerous examples whereby misexpression of a murine gene in *Xenopus *specifies distinct cell fates compared to misexpression in a mouse model (for example, *Mash1 *promotes a rod fate when misexpressed in mouse and a bipolar fate in *Xenopus *[59,60]. Moreover, in a previous study we showed that murine *Zac1 *unexpectedly promoted proliferation in *Xenopus *retina [26], in sharp contrast to its ability to promote cell cycle exit in the murine retina (this study) and cell lines *in vitro *[21,24]. To simplify our model of *Zac1 *retinal function, we therefore consider results obtained in mouse and *Xenopus *as independent systems where gene function may differ substantively.

### *Zac1 *regulates amacrine cell production cell non-autonomously

Previous studies based on ablation of mature amacrine cells [14] and aggregation of early progenitors with post-mitotic retinal cells [13] demonstrated that amacrine cell number is regulated by negative feedback, but the molecular mechanisms were unknown. Using similar aggregation assays, we showed that *Zac1 *is required in postnatal retinal cells to limit the number of amacrine cells generated [14]. With the exception of rods, numbers of all other retinal cells were not grossly perturbed in *Zac1 *mutants. The loss of amacrine cell negative feedback therefore does not affect later-born cell types, consistent with previous cell aggregation experiments [1,13]. We thus propose a model whereby initial reductions in amacrine cell genesis, beginning at E16 in wild-type retinae, occurs when progenitors switch to the next competence window to make later-born rods, bipolar cells and Müller glia, an event that is *Zac1*-independent. This would be followed early postnatally by *Zac1/TGFβII*-regulated feedback inhibition serving as the final signal to halt amacrine cell genesis (Figure 9).

Feedback pathways exist in diverse biological systems, including the counting factor in *Dictyostelium*, which dictates group size [2], *Drosophila miRNA9a*, which regulates sensory organ precursor number by downregulating *Senseless *expression [61], and the well established role of feedback signals in regulating cell number in vertebrate liver, pancreas, olfactory epithelium and retina [2]. Feedback pathways operate by secreting limiting amounts of extrinsic signals that must reach threshold levels to signal cessation of cell genesis [2]. Our data support the idea *Zac1 *acts in post-mitotic amacrine cells during the postnatal period to regulate TGFβII expression, which in turn suppresses amacrine cell genesis. However, our analysis of TGFβRII mutants also indicates that deleting TGFβ signaling earlier in development (that is, from E16 to E18.5), during the peak period of amacrine cell genesis, can also influence amacrine cell genesis. Invoking a threshold model for TGFβII could help explain why defects in cell cycle exit and expansion of the amacrine cell population were not completely penetrant phenotypes in *Zac1 *mutants. Indeed, developmental processes are known to be highly sensitive to levels of signaling molecules, and stochastic differences in signaling often account for phenotypic variability [62]. Moreover, abrogation of the feedback pathway regulating sense organ production in *Drosophila*, through deletion of *miR-9a*, similarly results in variable expressivity and penetrance of neuronal overproduction [61].

Notably, amacrine cell migration defects and the subsequent formation of an ECL were independent of attenuated TGFβ signaling in *Zac1 *mutant retinae. While we attribute the generation of an ECL to the mutation of *Zac1*, it remains a possibility that ECL formation requires both this genetic deletion as well as the loss of RGCs that occurs in retinal explant cultures, a possibility we cannot directly address given that *Zac1 *mutants die at birth. Another possibility is that *Zac1 *directly regulates cell migration by controlling the expression of cell adhesion genes, an idea based on a meta-analysis of microarray data in which several extracellular matrix molecules that could potentially modulate cell adhesion/migration were found to be co-regulated with *Zac1 *[23]. The underlying cause of ECL formation is the subject of current investigations.

## Conclusion

Here we demonstrate that *Zac1 *is an essential negative regulator of retinal size, controlling the absolute number of rod and amacrine cells generated during development. Strikingly, while *Zac1 *acts as a direct negative regulator of a rod fate, it negatively regulates amacrine cell genesis via TGFβII-mediated negative feedback inhibition. *Zac1 *and *TGFβII *are thus the first tumor surveillance molecules shown to control neuronal number through a negative feedback, 'cell sensing' mechanism. In summary, *Zac1 *regulates cell number and migration in the developing retina, highly reminiscent of its function in the prevention of tumor formation, suggesting that similar cellular and molecular mechanisms may underlie these processes.

## Materials and methods

### Animals and genotyping

For embryo staging, the day of the vaginal plug was considered E0.5. Generation of the *Zac1 *mutant allele was previously described [23]. The *Zac1 *mutant allele was maintained on a C57BL/6 background. *Zac1*^+m/- ^heterozygous embryos were generated by crossing *Zac1*^+/- ^heterozygous males to C57BL/6 females. Primers for PCR genotyping (35 cycles; 94° for 1 minute, 60° for 1 minute, and 72° for 1 minute) of *Zac1 *were: wild type 5': AGTGACTCCCCACCTTCTTTCTG; wild type 3': CTTGCCACATTTTTGACAGCG; mutant 5': TGACCGCTTCCTCGTGCTTTAC; mutant 3': CCCCCCAGAATAGAATGACACC. Genotyping of GLAST::CreERT2 and R26R reporter mice were previously described [46]. The floxed TGFβRII allele was previously reported [45] and was genotyped by PCR (38 cycles; 95° for 30 s, 62° for 30 s, and 72° for 40 s) with: primer 1 5': TGG GGATAGAGGTAGAAAGACATA-3'; primer 2 5': TATGGACTGGCT TTTGTATTC. To induce deletion of the TGFβRII gene, 3 mg of tamoxifen was administered by oral gavage at E16.0 as previously described [46].

### RNA *in situ *hybridization

For RNA *in situ *hybridization, tissue preparation and experimental procedures were followed as previously described [25]. Briefly, tissue was fixed in 4% paraformaldehyde (PFA)/1X-phosphate buffered saline (PBS) overnight at 4°C, cryopreserved in 20%sucrose/1X PBS overnight at 4°C and embedded in Cryomatrix™ (Anatomical Pathology USA [Pittsburgh, PA, USA]). Digoxygenin (dig)-labelled probes were generated using a dig-UTP labeling mix and T3, T7 or SP6 RNA polymerases according to the manufacturer's instructions (Roche [Laval, QC, Canada]). Mouse probes included *Zac1 *[25], *Hes1*, *Hes5, Mash1 *[63], *Ngn2 *[64], *Math3 *[65], *Math5 *[60], *NeuroD *[66], *Pax6 *[67], *Rx *[68], *Crx *[69], *Chx10 *[70]. *Foxn4 *[42] and *Barhl2 *[71], *Six3 *[41] and *s-opsin *[72].

### Immunohistochemistry and histochemistry

For immunohistochemistry, fixation in 4% PFA/1 × PBS was shortened to 1–2 h at 4°C. Primary antibodies were incubated on slides overnight at 4°C or 1 h at room temperature. The following primary antibodies were used: rabbit active-caspase 3 (1/500; Promega [Madison, WI, USA]), mouse Brn3a (1/500; Chemicon [Temecula, CA, USA]), goat anti-Brn3b (1/250; Santa Cruz [Santa Cruz, CA, USA]), mouse anti-BrdU (5-bromo-2'-deoxyuridine, 1/500; Roche), rat-anti-BrdU (1/10; Oxford Biotech [now Antibodies by Design, Raleigh, NC, USA]), rabbit anti-calbindin (1/1,000; SWANT [Bellinzona, Switzerland]), mouse anti-cyclinD1 (1/100; Santa Cruz), rabbit anti-Chx10 (1/50; Rod McInnes), mouse anti-CRALBP (1/5,000; Jack Saari), rabbit anti-GFP (1/500, Chemicon), goat anti-Math3 (1/100, Santa Cruz), mouse anti-neurofilament 200 (1/500; NF200, Sigma [Oakville, ON, Canada]), rabbit anti-Nr2e3 (1/100; Chemicon), rabbit anti-Pax6 (1/500; Babco [Richmond, CA, USA]]), mouse anti-Pax6 (1/4, Developmental Studies Hybridoma Bank [Iowa City, IA, USA]), rabbit anti-p27^Kip1 ^(1/500; NeoMarker Lab Vision, [Freemont, CA, USA] ]), mouse anti-protein kinase C (PKC; 1/500; Sigma), mouse anti-rhodopsin (1/500; Chemicon), mouse anti-syntaxin (1/2,000; Sigma), rabbit anti-TGFβII (1/100; Santa Cruz), rabbit anti-TGFβRI (1/100; Santa Cruz), rabbit anti-TGFβRII (1/100; Santa Cruz), rabbit anti-phospho-Smad2/3 (1/100; Santa Cruz), guinea pig anti-GLAST (1/8,000; Chemicon) and rabbit anti-Zac1 (1/1,000 [24]). Primary antibodies were washed 3 times in PBS with 0.1% triton X-100 (PBT) and detected using secondary antibodies conjugated with Cy3- (1/500; Jackson ImmunoResearch Laboratories, Inc. [West Grove, PA, USA]) or Alexa488 (1/500; Molecular Probes [Invitrogen, Eugene, OR, USA]). Secondary antibodies were diluted in PBT and left on the slides for 1 h prior to 3–10 minute washes with PBT. Note that the TSA™ Tyramide-Fluorescein Immunostaining Kit (NEL701, Perkin-Elmer [Shelton, CT, USA]) was used to amplify anti-TGFβII, TGFβRI, TGFβRII and phospho-Smad2/3 immunostaining as per the manufacturer's instructions. Peanut Agglutinin (PNA) staining was carried out using a 1:200 dilution of the PNA lectin incubated at 37°C for 30 minutes. Sections were then stained for five minutes with DAPI, washed an additional three times with PBS, and mounted with AquaPolymount. β-Galactosidase activity was detected using X-gal as a substrate as previously described [73].

### BrdU labeling

To label S-phase progenitors, pregnant females were injected intraperitoneally with 100 μg/g body weight BrdU (Sigma) 30 minutes prior to sacrifice. For birthdating studies, BrdU was added to the culture media at a final concentration of 10 μM. Embryos were processed for anti-BrdU staining as above except for the addition of a pretreatment with 2N HCl for 30 minutes at 37°C. BrdU immunolabeling after RNA *in situ *hybridization was carried out using 3,3'-diaminobenzidine (DAB) as a substrate using the Vectastain kit (Vector Laboratories Inc. [Burlingame, CA, USA]).

### Western blotting

Retinae were lysed for 15 minutes on ice in RIPA buffer (1% SDS, 1% sodium deoxycholate, 0.1% Nonidet P-40 in 50 mM Tris-HCl (pH 7.6)/150 mM NaCl) plus protease (Complete inhibitor tablet, Roche) and phosphatase (5 mM NaF and 1 mM orthovanadate) inhibitors. Cell lysates were cleared and protein concentrations determined via Bradford analysis. Cell free extract (25 μg) was loaded per lane on a 12% (Smad/phospho-Smad) or 15% (TGFβII) SDS-PAGE gel. Protein was then transferred to PVDF membrane at 80 V for 1 h. Membranes were blocked in 5% skim milk powder or 5% bovine serum albumin (for phospho-Smad) in tris-buffered saline with 0.1% tween 20 (TBST) and then incubated with anti-phospho-Smad2/3 (1/200; Santa Cruz), Smad2/3 (1/200; Santa Cruz), TGFβII (1/200; Santa Cruz) or anti-β-actin (1/5,000, AbCam [Cambridge, MA, USA]) overnight at 4°C. Membranes were washed three times for ten minutes each prior to incubation in horse radish peroxidase (HRP)-conjugated secondary antibodies and development with ECL (Roche).

### Retinal explants

Retinae were dissected and grown as explants as previously described [74]. Briefly, the retinal pigmented epithelium (RPE) and lens were removed from dissected eyes, and the retina was flattened and cultured GCL-up on a Nucleopore Track-Etch membrane (13 mm; Whatman [Maldstone, England]) in explant media (50% MEM, 25% Hanks Solution, 25% horse serum, 6.75 mg/ml glucose, 200 μM L-glutamine, 2.5 mM HEPES) at 37°C in 5% CO_2_. The TGFβRII-Fc soluble receptor inhibitor (R&D systems [Burlington, ON, Canada]) was added at 0.5 μg/ml dissolved in PBS (vehicle control) every second day as described [44]. Recombinant TGFβII (R&D systems) was added to explants every second day at 1 ng/ml.

### Aggregation assays

Retinae were dissected, dissociated into single cell suspensions and cultured as aggregates essentially as described [13,75]. Briefly, E14.5 wild-type retinae were dissociated in trypsin (10 min/37°C) and triturated in DMEM/10% fetal calf serum with 100 μl DNAseI (2 mg/ml). Dissociated progenitors were labeled in media with 10 μM BrdU for 1 h. BrdU was washed out and cells were resuspended in culture media at 5 × 10^5 ^cells/ml. For co-cultures, 100 μl (5 × 10^4 ^cells) of labeled E14.5 progenitors were added to a 20-fold excess (1 × 10^6 ^cells) of dissociated E18.5 wild-type or *Zac1 *mutant cells. Aggregated cells were pelleted by centrifuging for 8 minutes at 2,200 rpm and pellets were transferred after 1 h onto Nucleopore membranes and cultured 8DIV. Pellets were then dissociated and plated on poly-D-lysine-coated slides for immunostaining.

### Retinal electroporation

For misexpression, full-length *Zac1 *cDNA [26] was cloned into a pCIG2 expression vector containing a CMV-enhancer/chicken β-actin promoter and IRES-EGFP cassette (gift from Franck Polleux) [76]. For electroporation, eyes were dissected and the RPE removed prior to immersion in 10 μl DNA (3 μg/μl) on a 3% agarose gel plug. Platinum electrodes were placed on either side of the eye (E15.5, 4 mm spacing; and E18.5, 5 mm spacing) and seven 20 ms pulses of 25 V were applied. Electroporated retinae were then cultured as explants.

### Cell counts and statistical analysis

Immunoreactive cells were counted in sections adjacent to the optic nerve or site of optic nerve transection in explants. In all experiments, cells were counted from a minimum of three embryos (or explants) and three sections per embryo (or explant). The total number of individual retinae analyzed per experiment (n values) is presented in the results section and the total number of cells counted per experiment is presented in the figure legends. All quantification was done from photomicrographs representing a 0.33 mm × 0.25 mm counting field. Statistical variation was determined using the standard error of the mean (SEM). Statistical significance was calculated using a Student's *t*-test, individually comparing experimental bars against wild-type or control counts.

## Additional data files

The following additional data are available with the online version of this paper. Additional data file 1 is a figure showing that Zac1 is expressed in dividing progenitors at embryonic stages and differentiated cells at postnatal stages. Additional data file 2 is a figure showing *Zac1 *genotyping and verification of maternal imprinting in the embryonic retina. Additional data file 3 is a figure showing that equivalent numbers of bipolar cells, Müller glia, horizontal cells and cone photoreceptors develop in wild-type and *Zac1 *mutant retinal explants, while the number of amacrine cells increased in *Zac1 *mutant retinae. Additional data file 4 is a figure showing that RGC differentiation is unperturbed in *Zac1-*deficient retinae at E18.5. Additional data file 5 is a figure showing that amacrine cell precursors do not undergo more apoptosis or divide ectopically in *Zac1 *mutant retinae. Additional data file 6 is a figure showing that misexpression of *Zac1 *in the retina does not affect amacrine cells genesis. Additional data file 7 is a figure showing that the molecular profile of *Zac1*-deficient retinal progenitors is unperturbed at E18.5. Additional data file 8 is a figure showing that amacrine cell marker expression domains are expanded in E18.5 *Zac1 *mutant retinal explants cultured 4 DIV.

## Competing interests

The author(s) declare that they have no competing interests.

## Authors' contributions

LM carried out the vast majority of the experiments with technical assistance from NK. RC carried out western blot analysis, apoptosis studies and TGFβRII conditional knock-out analysis. AV generated *Zac1 *knock-out mice in the laboratory of LJ, who also provided *Zac1 *antiserum and comments on the manuscript. DC generated TGFβRII conditional knock-out embryos in the laboratory of MG. SMF provided intellectual input and comments on the manuscript. The experiments were primarily designed by LM and CS.

## Supplementary Material

Additional data File 1 Zac1 is expressed in dividing progenitors at embryonic stages and differentiated cells at postnatal stages. **(a-c) **E15.5 retinae co-immunolabeled with anti-Zac1 (red, a,c) and anti-syntaxin (green, b) and merged image (c). **(d-f) **E15.5 retinae co-immunolabeled with anti-Zac1 (red, d) and anti-BrdU (green, e) and merged image (f). **(g,h) **Expression of *Zac1 *transcripts (g) and protein (h) in P21 retinae. Click here for file

Additional File 2 *Zac1 *genotyping and verification of maternal imprinting in the embryonic retina. **(a) **PCR genotyping of wild-type and *Zac1 *mutant alleles. **(b,c) **Zac1 immunostaining of E15.5 wild-type and *Zac1*^+m/- ^mutant retinae revealed a loss of expression in heterozygous embryos carrying a maternal wild-type allele. Click here for file

Additional data File 3 Equivalent numbers of bipolar cells, Müller glia, horizontal cells and cone photoreceptors develop in wild-type and *Zac1 *mutant retinal explants, while the number of amacrine cells increased in *Zac1 *mutant retinae. E18.5 wild-type **(a,c,e,g,i,k,m,o,q) **and *Zac1*-deficient **(b,d,f,h,j,l,n,p,r) **retinae were cultured 8DIV and labelled with Chx10 (red, a,b) for bipolar cells, p27^Kip1 ^(red, c,d) and CRALBP (red, e,f) for Müller glia, peanut agglutinin (PNA, green, g,h) and *s-opsin *(i,j) for cones, Bhlhb5 (green, k,l) for GABAergic amacrine cells, calbindin for horizontal cell bodies (red, with processes in outer plexiform layer; m,n) and AII amacrine cells (deeper in INL; m,n), and GABA (red, o,p) and GlyT1 (red, q,r) for amacrine cell subtypes. Explants were counterstained with DAPI (blue). Click here for file

Additional data File 4 RGC differentiation is unperturbed in *Zac1-*deficient retinae at E18.5. Brn3a **(a,b) **and Brn3b **(c,d) **immunolabeling of RGCs in wild-type (a,c) and *Zac1 *mutant (b,d) retinae at E18.5. Quantitation of Brn3a **(e) **and Brn3b **(f) **expressing cells revealed equivalent numbers of RGCs in wild-type (n = 3 retinae; black bar) and *Zac1 *mutant (n = 3 retinae; white bar) retinae. Brn3a (p = 0.95; wild-type: 6.4 ± 1.0% retinal cells; 774 Brn3a^+^/12138 DAPI^+^; *Zac1 *mutant: 7.4 ± 0.4%; 743 Brn3a^+^/10123 DAPI^+^) and Brn3b (p = 0.23; wild-type: 3.3 ± 0.7%; 269 Brn3b^+^/6813 DAPI^+^; *Zac1 *mutant: 5.0 ± 0.7%; 393 Brn3b^+^/7818 DAPI^+^). Click here for file

Additional data File 5 Amacrine cell precursors do not undergo more apoptosis or divide ectopically in *Zac1 *mutant retinae. **(a-d) **E10.5 (a,b) and E15.5 (c,d) retinae immunostained for activated caspase-3 (ac-3) (red) in wild-type (a,c) and *Zac1*^+m/-^(b,d) embryos. Inserts in c,d are high magnification images of ac-3^+ ^cells. **(e-h) **Ac-3 (red)/Pax6 (green) double^+ ^cells label apoptotic amacrine cells in E18.5→4DIV explants. g and h are high magnification images of boxed areas in e and f, respectively. Ac-3^+ ^amacrine cells are marked by arrowheads (g,h). **(i) **Percentage of Pax6^+^/ac-3^+ ^apoptotic amacrine cells in wild-type (black bars; 45 ac-3/Pax6 double^+^/2925 Pax6^+^) and *Zac1*^+m/- ^(white bars; 22 ac-3/Pax6 double^+^/2538 Pax6^+^) E18.5→4DIV explants. **(j) **Percentage of BrdU^+^/Pax6^+ ^dividing amacrine cells in total Pax6^+ ^population in E18.5 explants cultured 1DIV, 2DIV and 4DIV. 1DIV (p = 0.40; wild-type: 1.1 ± 0.3%; n = 3 explants; 12 BrdU/Pax6 double^+^/1071 Pax6^+^; *Zac1 *mutant: 0.7 ± 0.2%; n = 3 explants; 10 BrdU/Pax6 double^+^/1386 Pax6^+^), 2DIV (p = 0.76; wild-type: 0.9 ± 0.3%; n = 3 explants; 16 BrdU/Pax6 double^+^/1698 Pax6^+^; *Zac1 *mutant: 0.8 ± 0.3%; n = 3 explants; 16 BrdU/Pax6 double^+^/1983 Pax6^+^) and 4DIV (p = 0.44; wild-type: 0.2 ± 0.2%; n = 3 explants; 5 BrdU/Pax6 double^+^/712 Pax6^+^; *Zac1 *mutant: 0.4 ± 0.3%; n = 3 explants; 16 BrdU/Pax6 double^+^/2307 Pax6 single^+^). Blue is DAPI counterstain. Click here for file

Additional data File 6 Misexpression of *Zac1 *in the retina does not affect amacrine cells genesis. **(a-f) **P0 retinae were electroporated with control pCIG2 (a,c,e) or pCIG2-*Zac1 *(b,d,f) and cultured 8DIV. Electroporated cells were detected by GFP epifluorescence (green; a,b) and amacrine cells were identified by anti- syntaxin (red; c,d). (e,e',f,f') Merged images show similar numbers of GFP-positive electroporated cells that expressed syntaxin (Syn) after control (e) and *Zac1 *(f) electroporations. Arrowheads indicate electroporated cells that differentiated into amacrine cells. e' and f' are high magnification images of boxed area in e and f. **(g) **Quantitation of the percentage of electroporated cells that differentiate into amacrine cells after control pCIG2 (black bar; n = 3) or pCIG2-*Zac1 *(white bar; n = 3) electroporations. pCIG2 at E15.5: 62.3 ± 6.5%; 519 syntaxin/GFP double^+^/787 GFP^+^; *Zac1 *at E15.5: 72.7 ± 5.4%; 379 syntaxin/GFP double^+^/680 GFP^+^; pCIG2 at E17.5: 39.3 ± 5.4%; 897 syntaxin/GFP double^+^/2520 GFP^+^; *Zac1 *at E17.5: 51.3 ± 2.0%; 456 syntaxin/GFP double^+^/928 GFP^+^; pCIG2 at P0: 11.7 ± 3.4%; 81 syntaxin/GFP double^+^/552 GFP^+^; *Zac1 *at P0: 10.2 ± 2.3%; 83 syntaxin/GFP double^+^/376 GFP^+^. Click here for file

Additional data File 7 Molecular profile of *Zac1*-deficient retinal progenitors is unperturbed at E18.5. RNA in situ hybridization of E18.5 wild-type (non-prime) and *Zac1*-deficient (prime) retinae with *Hes5 ***(a,a')**, *Hes1 ***(b,b')**, *Rx ***(c,c')**, *Chx10 ***(d,d')**, *Crx ***(e,e')**, *Barhl2 ***(f,f')**, *Mash1 ***(g,g')**, *Foxn4 ***(h,h')**, *NeuroD ***(i,i')**, *Math3 ***(j,j')**, *Math5 ***(k,k') **and *Pax6 ***(l,l') **probes. Click here for file

Additional data File 8 Amacrine cell marker expression domains are expanded in E18.5 *Zac1 *mutant retinal explants cultured 4 DIV. Marker expression in E18.5 retinal explants cultured 4 DIV from wild-type (a,c,e,g,i,k,m) and *Zac1*-deficient (b,d,f,h,j,l,n) embryos. *Chx10 *transcript **(a,b) **and Chx10 protein **(c,d) **distribution in retinal explants. *Crx ***(e,f)**, *Hes1 ***(g,h)**,*Six3 ***(i,j), ***Barhl2 ***(k,l) **and *Math3***(m,n) **expression. Explants processed for *Hes1 *and *Six3 *RNA in situ hybridization were also immunolabeled with anti-BrdU (after 30 min exposure) to label dividing cells. Click here for file
