# Supplementary figures and images for: Zac1 functions through TGFβII to negatively regulate cell number in the developing retina
Source: Neural Dev. 2007 Jun 8;2:11. doi: 10.1186/1749-8104-2-11 (PMC1913510; doi:10.1186/1749-8104-2-11)

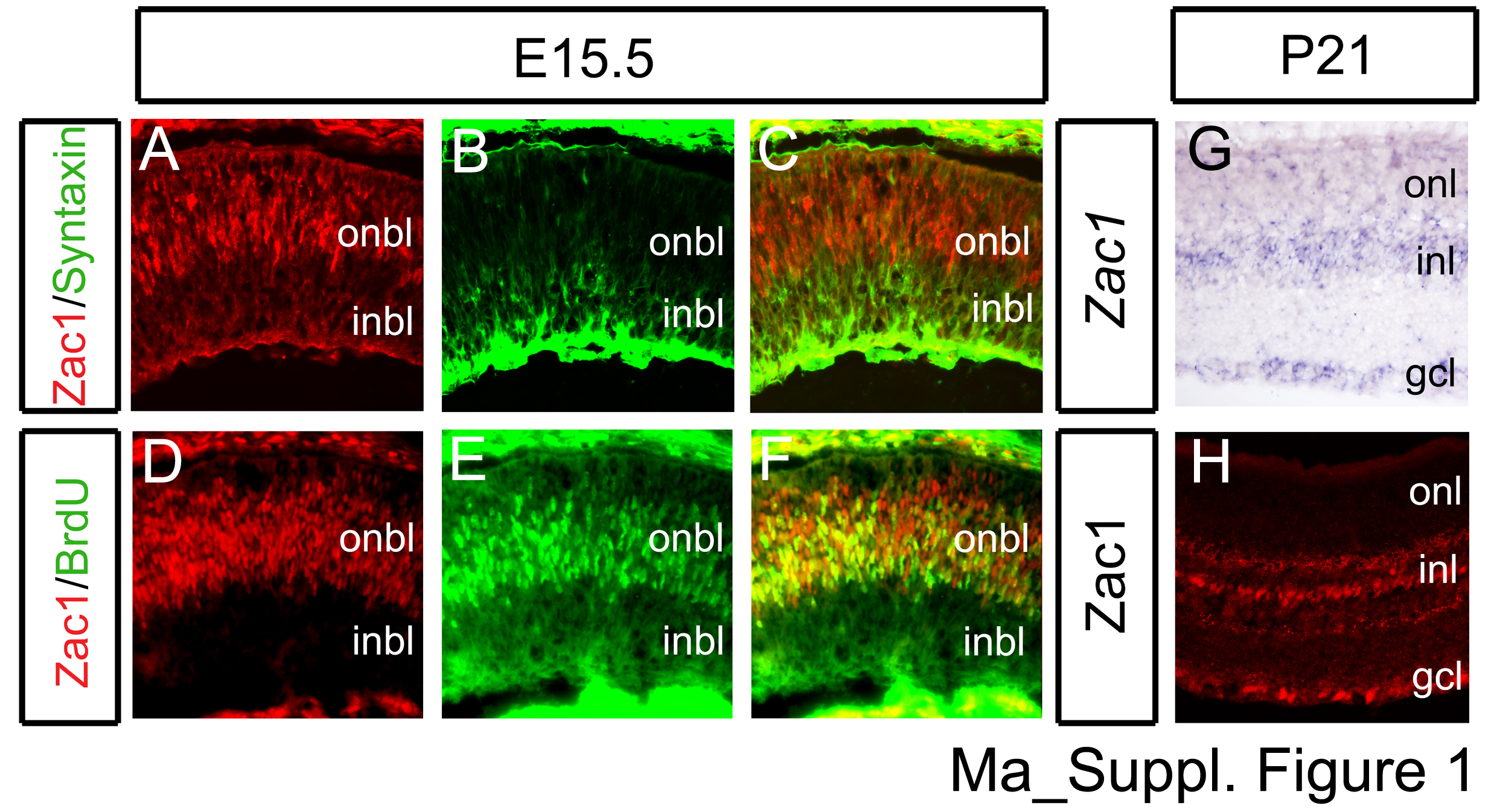

Supplement: Additional data File 1 — Zac1 is expressed in dividing progenitors at embryonic stages and differentiated cells at postnatal stages. (a-c) E15.5 retinae co-immunolabeled with anti-Zac1 (red, a,c) and anti-syntaxin (green, b) and merged image (c). (d-f) E15.5 retinae co-immunolabeled with anti-Zac1 (red, d) and anti-BrdU (green, e) and merged image (f). (g,h) Expression of Zac1 transcripts (g) and protein (h) in P21 retinae. [file 1749-8104-2-11-S1.jpeg]

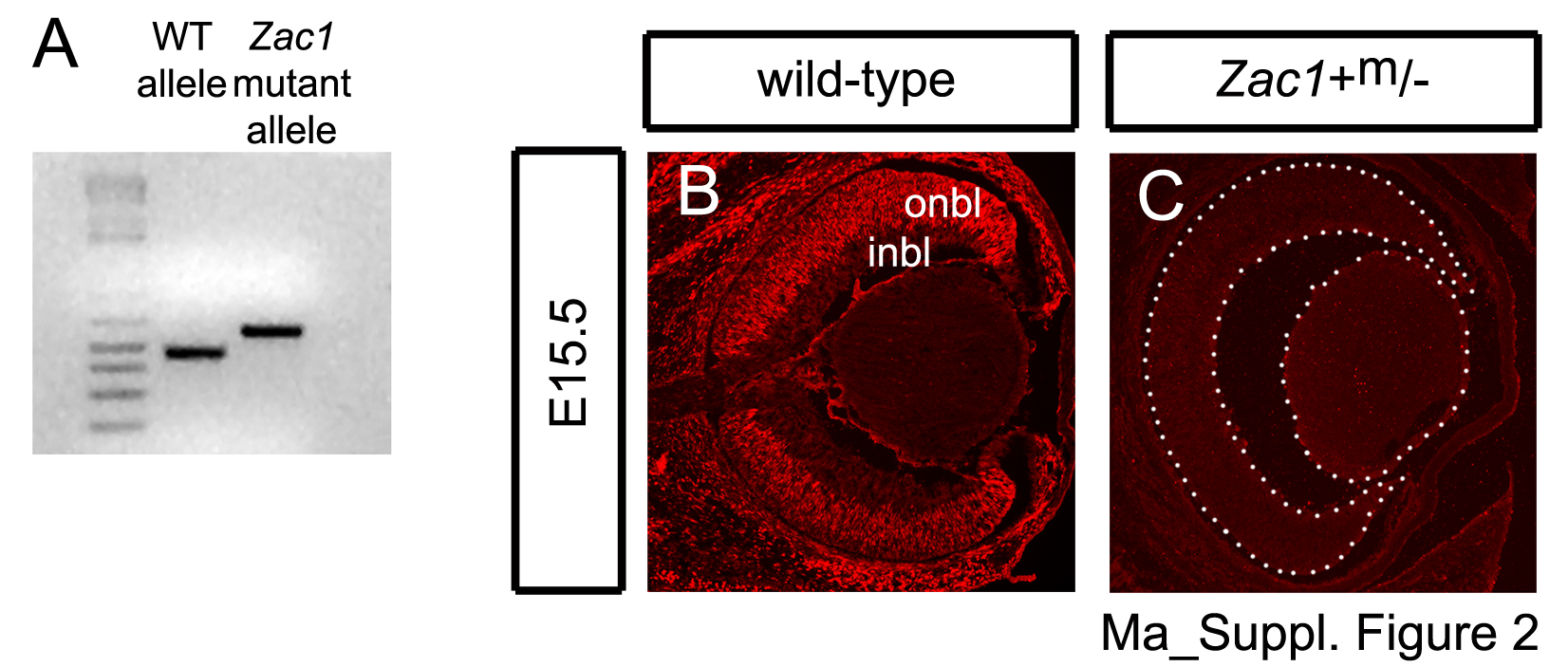

Supplement: Additional File 2 — Zac1 genotyping and verification of maternal imprinting in the embryonic retina. (a) PCR genotyping of wild-type and Zac1 mutant alleles. (b,c) Zac1 immunostaining of E15.5 wild-type and Zac1+m/- mutant retinae revealed a loss of expression in heterozygous embryos carrying a maternal wild-type allele. [file 1749-8104-2-11-S2.jpeg]

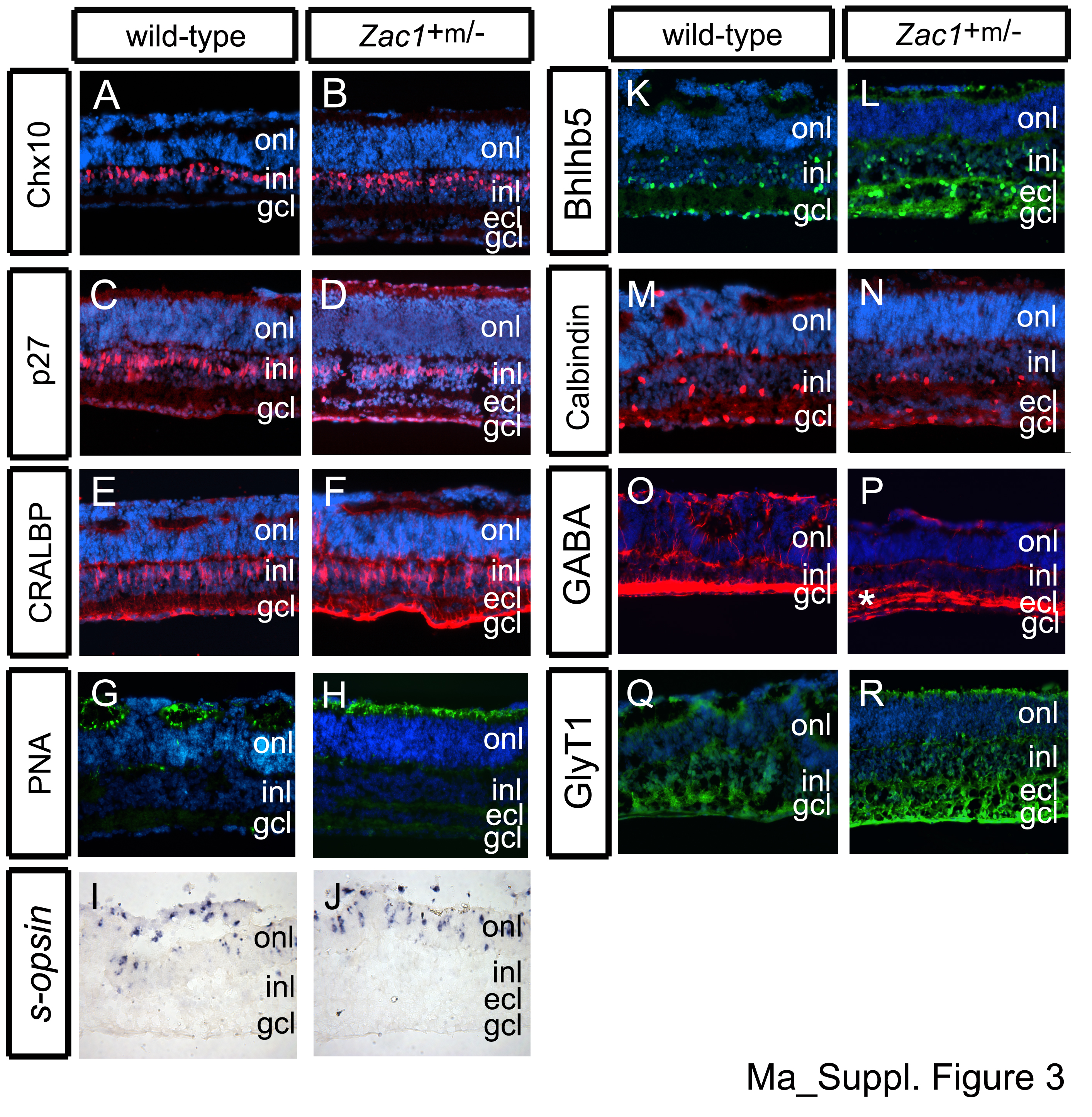

Supplement: Additional data File 3 — Equivalent numbers of bipolar cells, Müller glia, horizontal cells and cone photoreceptors develop in wild-type and Zac1 mutant retinal explants, while the number of amacrine cells increased in Zac1 mutant retinae. E18.5 wild-type (a,c,e,g,i,k,m,o,q) and Zac1-deficient (b,d,f,h,j,l,n,p,r) retinae were cultured 8DIV and labelled with Chx10 (red, a,b) for bipolar cells, p27Kip1 (red, c,d) and CRALBP (red, e,f) for Müller glia, peanut agglutinin (PNA, green, g,h) and s-opsin (i,j) for cones, Bhlhb5 (green, k,l) for GABAergic amacrine cells, calbindin for horizontal cell bodies (red, with processes in outer plexiform layer; m,n) and AII amacrine cells (deeper in INL; m,n), and GABA (red, o,p) and GlyT1 (red, q,r) for amacrine cell subtypes. Explants were counterstained with DAPI (blue). [file 1749-8104-2-11-S3.jpeg]

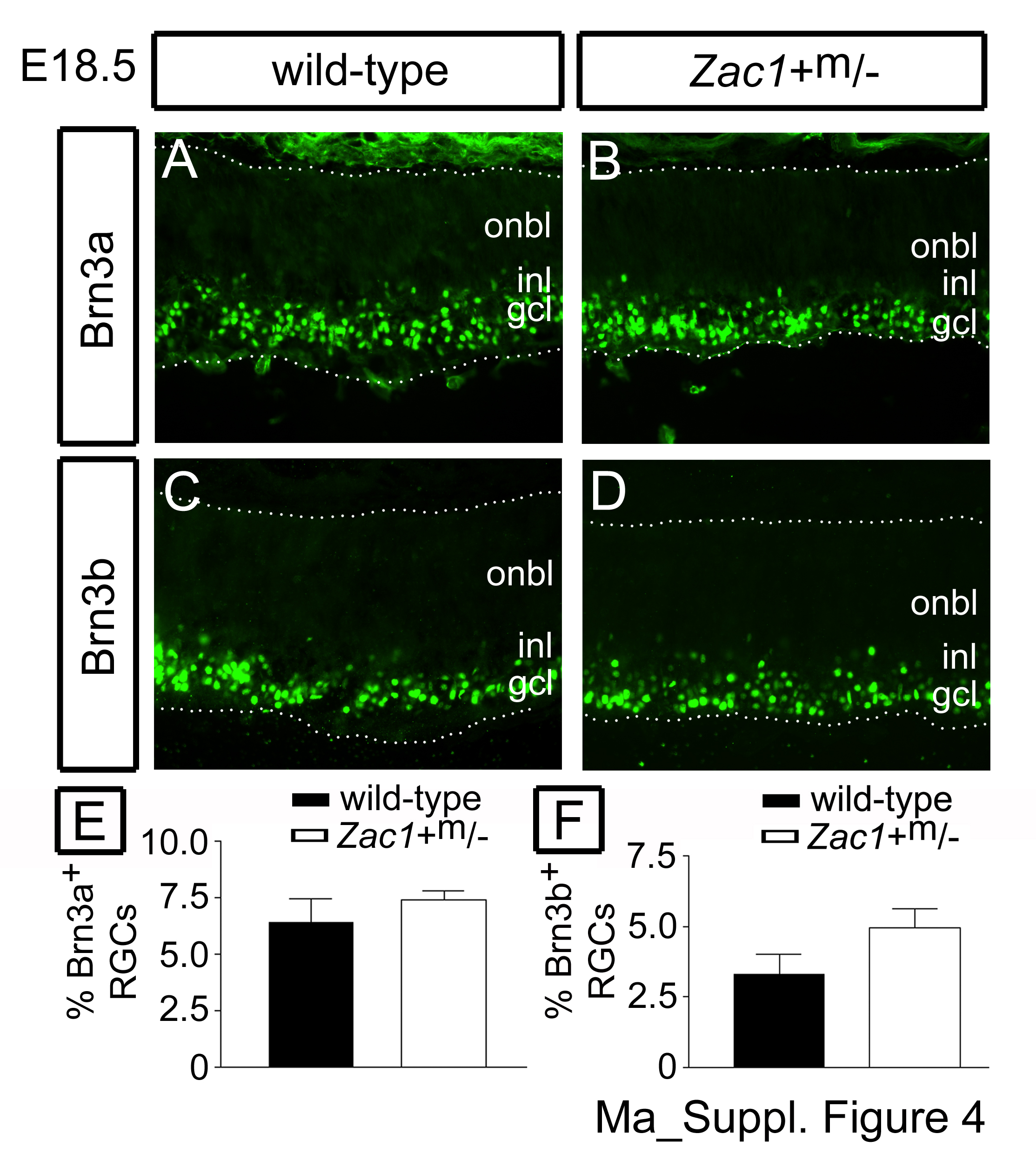

Supplement: Additional data File 4 — RGC differentiation is unperturbed in Zac1-deficient retinae at E18.5. Brn3a (a,b) and Brn3b (c,d) immunolabeling of RGCs in wild-type (a,c) and Zac1 mutant (b,d) retinae at E18.5. Quantitation of Brn3a (e) and Brn3b (f) expressing cells revealed equivalent numbers of RGCs in wild-type (n = 3 retinae; black bar) and Zac1 mutant (n = 3 retinae; white bar) retinae. Brn3a (p = 0.95; wild-type: 6.4 ± 1.0% retinal cells; 774 Brn3a+/12138 DAPI+; Zac1 mutant: 7.4 ± 0.4%; 743 Brn3a+/10123 DAPI+) and Brn3b (p = 0.23; wild-type: 3.3 ± 0.7%; 269 Brn3b+/6813 DAPI+; Zac1 mutant: 5.0 ± 0.7%; 393 Brn3b+/7818 DAPI+). [file 1749-8104-2-11-S4.jpeg]

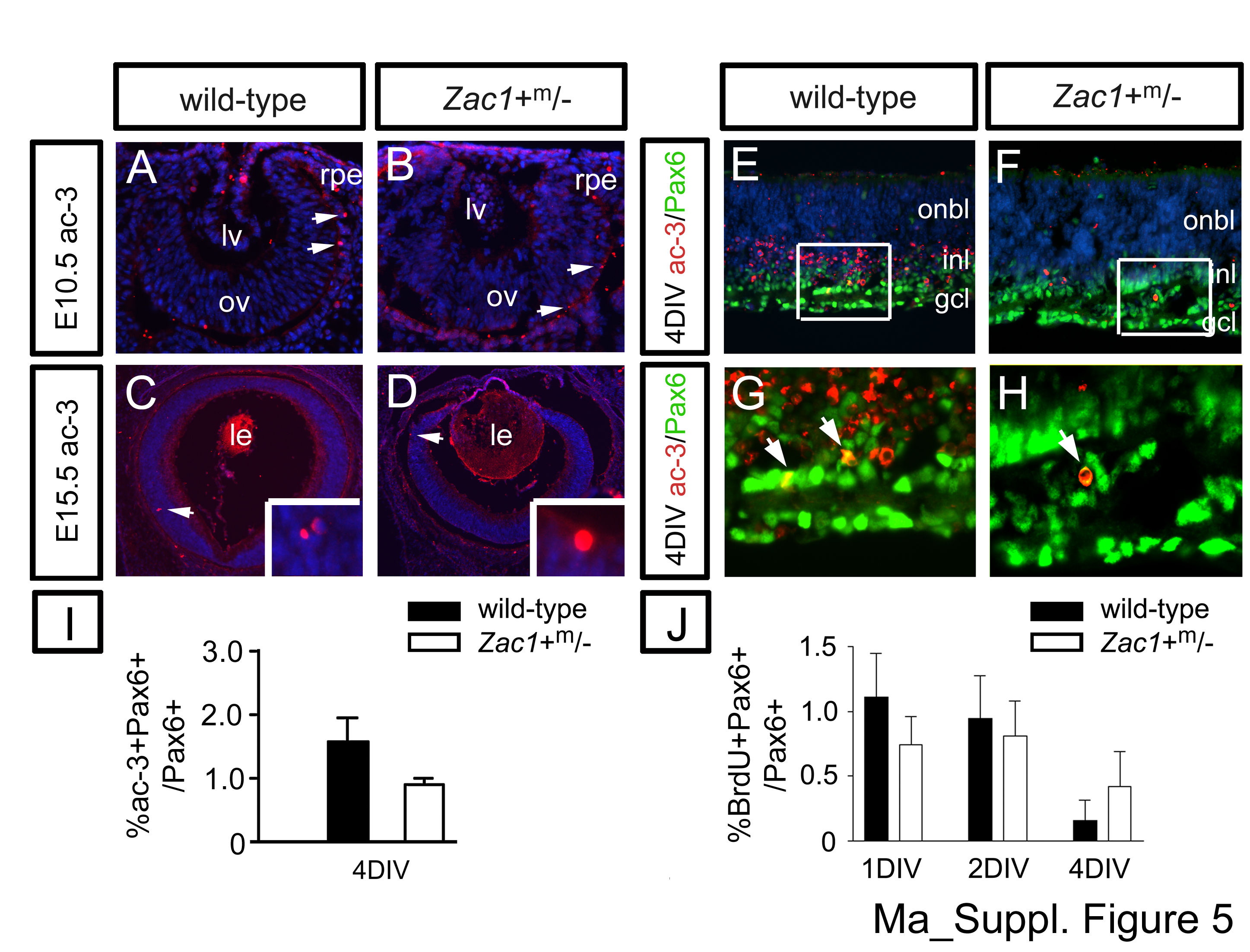

Supplement: Additional data File 5 — Amacrine cell precursors do not undergo more apoptosis or divide ectopically in Zac1 mutant retinae. (a-d) E10.5 (a,b) and E15.5 (c,d) retinae immunostained for activated caspase-3 (ac-3) (red) in wild-type (a,c) and Zac1+m/-(b,d) embryos. Inserts in c,d are high magnification images of ac-3+ cells. (e-h) Ac-3 (red)/Pax6 (green) double+ cells label apoptotic amacrine cells in E18.5→4DIV explants. g and h are high magnification images of boxed areas in e and f, respectively. Ac-3+ amacrine cells are marked by arrowheads (g,h). (i) Percentage of Pax6+/ac-3+ apoptotic amacrine cells in wild-type (black bars; 45 ac-3/Pax6 double+/2925 Pax6+) and Zac1+m/- (white bars; 22 ac-3/Pax6 double+/2538 Pax6+) E18.5→4DIV explants. (j) Percentage of BrdU+/Pax6+ dividing amacrine cells in total Pax6+ population in E18.5 explants cultured 1DIV, 2DIV and 4DIV. 1DIV (p = 0.40; wild-type: 1.1 ± 0.3%; n = 3 explants; 12 BrdU/Pax6 double+/1071 Pax6+; Zac1 mutant: 0.7 ± 0.2%; n = 3 explants; 10 BrdU/Pax6 double+/1386 Pax6+), 2DIV (p = 0.76; wild-type: 0.9 ± 0.3%; n = 3 explants; 16 BrdU/Pax6 double+/1698 Pax6+; Zac1 mutant: 0.8 ± 0.3%; n = 3 explants; 16 BrdU/Pax6 double+/1983 Pax6+) and 4DIV (p = 0.44; wild-type: 0.2 ± 0.2%; n = 3 explants; 5 BrdU/Pax6 double+/712 Pax6+; Zac1 mutant: 0.4 ± 0.3%; n = 3 explants; 16 BrdU/Pax6 double+/2307 Pax6 single+). Blue is DAPI counterstain. [file 1749-8104-2-11-S5.jpeg]

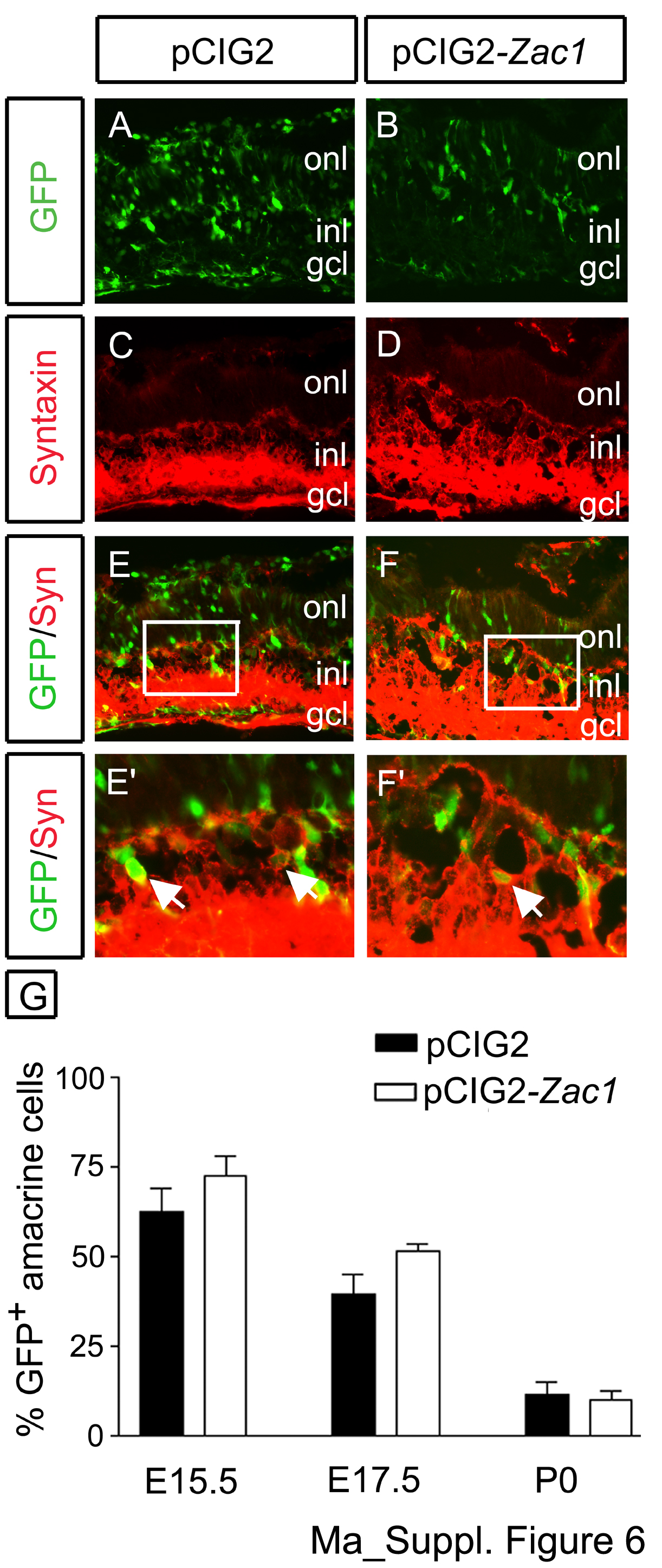

Supplement: Additional data File 6 — Misexpression of Zac1 in the retina does not affect amacrine cells genesis. (a-f) P0 retinae were electroporated with control pCIG2 (a,c,e) or pCIG2-Zac1 (b,d,f) and cultured 8DIV. Electroporated cells were detected by GFP epifluorescence (green; a,b) and amacrine cells were identified by anti- syntaxin (red; c,d). (e,e',f,f') Merged images show similar numbers of GFP-positive electroporated cells that expressed syntaxin (Syn) after control (e) and Zac1 (f) electroporations. Arrowheads indicate electroporated cells that differentiated into amacrine cells. e' and f' are high magnification images of boxed area in e and f. (g) Quantitation of the percentage of electroporated cells that differentiate into amacrine cells after control pCIG2 (black bar; n = 3) or pCIG2-Zac1 (white bar; n = 3) electroporations. pCIG2 at E15.5: 62.3 ± 6.5%; 519 syntaxin/GFP double+/787 GFP+; Zac1 at E15.5: 72.7 ± 5.4%; 379 syntaxin/GFP double+/680 GFP+; pCIG2 at E17.5: 39.3 ± 5.4%; 897 syntaxin/GFP double+/2520 GFP+; Zac1 at E17.5: 51.3 ± 2.0%; 456 syntaxin/GFP double+/928 GFP+; pCIG2 at P0: 11.7 ± 3.4%; 81 syntaxin/GFP double+/552 GFP+; Zac1 at P0: 10.2 ± 2.3%; 83 syntaxin/GFP double+/376 GFP+. [file 1749-8104-2-11-S6.jpeg]

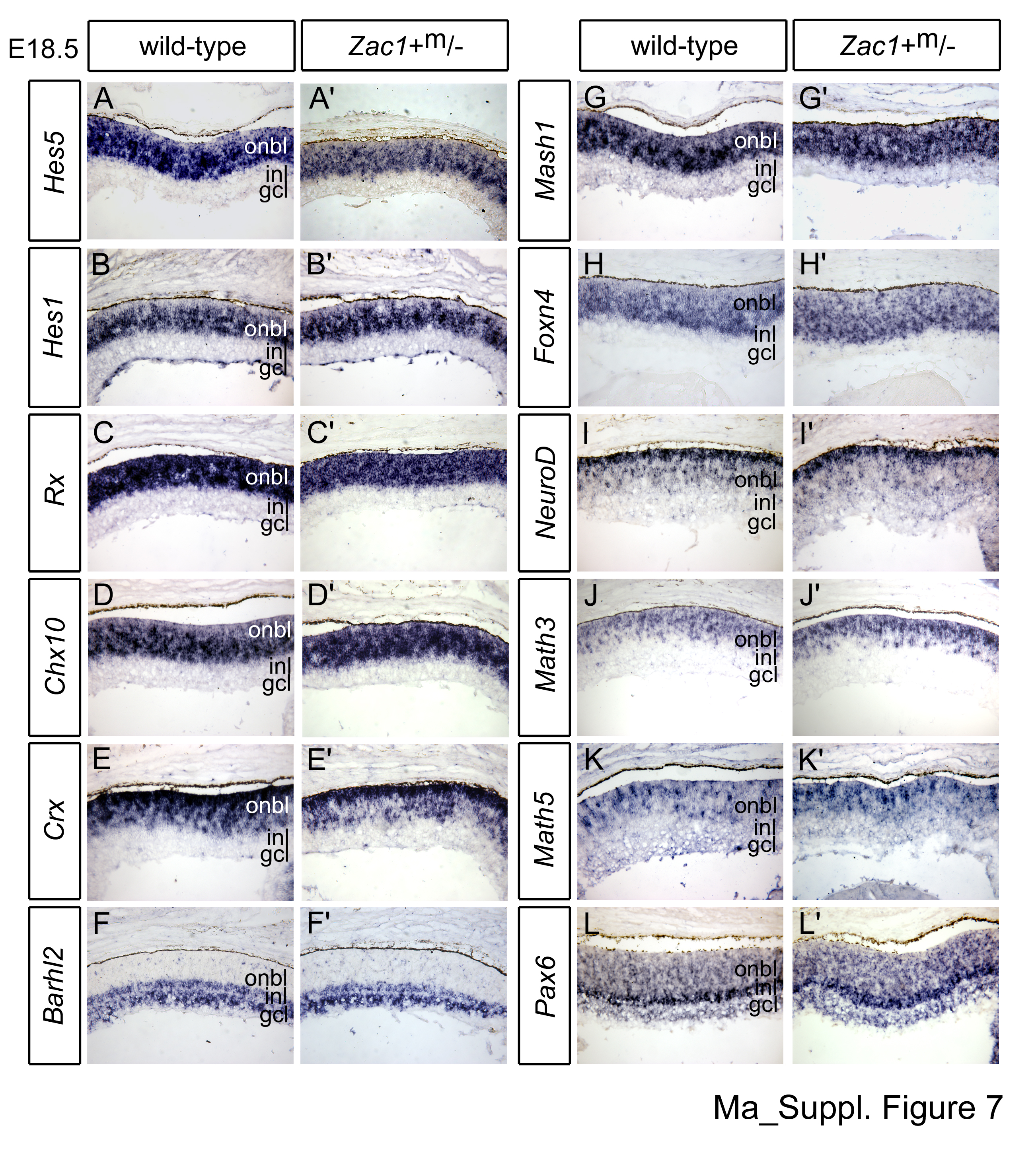

Supplement: Additional data File 7 — Molecular profile of Zac1-deficient retinal progenitors is unperturbed at E18.5. RNA in situ hybridization of E18.5 wild-type (non-prime) and Zac1-deficient (prime) retinae with Hes5 (a,a'), Hes1 (b,b'), Rx (c,c'), Chx10 (d,d'), Crx (e,e'), Barhl2 (f,f'), Mash1 (g,g'), Foxn4 (h,h'), NeuroD (i,i'), Math3 (j,j'), Math5 (k,k') and Pax6 (l,l') probes. [file 1749-8104-2-11-S7.jpeg]

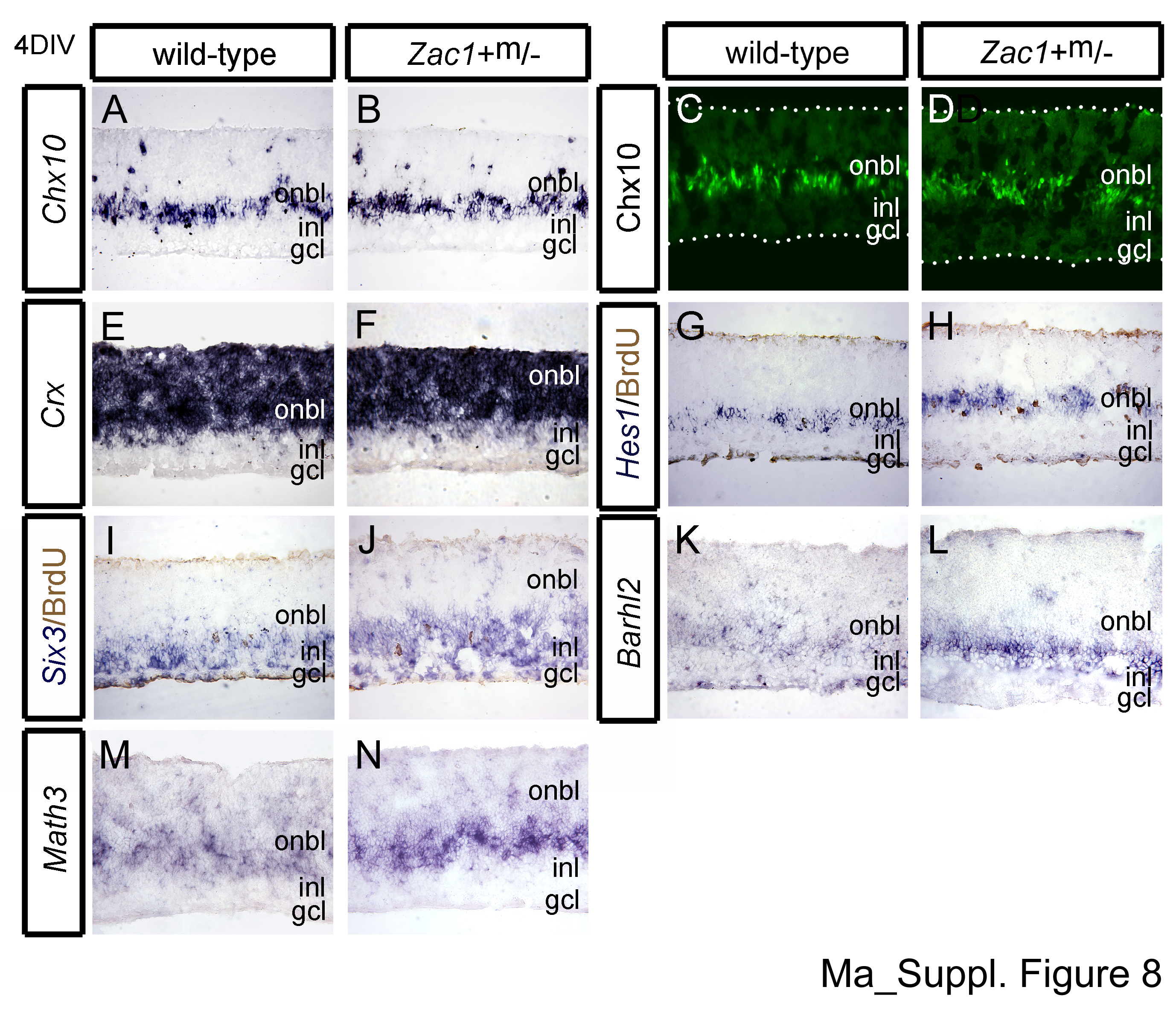

Supplement: Additional data File 8 — Amacrine cell marker expression domains are expanded in E18.5 Zac1 mutant retinal explants cultured 4 DIV. Marker expression in E18.5 retinal explants cultured 4 DIV from wild-type (a,c,e,g,i,k,m) and Zac1-deficient (b,d,f,h,j,l,n) embryos. Chx10 transcript (a,b) and Chx10 protein (c,d) distribution in retinal explants. Crx (e,f), Hes1 (g,h),Six3 (i,j), Barhl2 (k,l) and Math3(m,n) expression. Explants processed for Hes1 and Six3 RNA in situ hybridization were also immunolabeled with anti-BrdU (after 30 min exposure) to label dividing cells. [file 1749-8104-2-11-S8.jpeg]
